# Specific Inhibition of Phosphodiesterase-4B Results in Anxiolysis and Facilitates Memory Acquisition

**DOI:** 10.1038/npp.2015.240

**Published:** 2015-09-02

**Authors:** Alexander McGirr, Tatiana V Lipina, Ho-Suk Mun, John Georgiou, Ahmed H Al-Amri, Enoch Ng, Dongxu Zhai, Christina Elliott, Ryan T Cameron, Jonathan GL Mullins, Fang Liu, George S Baillie, Steven J Clapcote, John C Roder

**Affiliations:** 1 Department of Psychiatry, University of British Columbia, Vancouver, British Columbia, Canada; 2 Lunenfeld-Tanenbaum Research Institute, Mount Sinai Hospital, Toronto, Ontario, Canada; 3 Department of Medical Genetics, University of Toronto, Toronto, Ontario, Canada; 4 School of Biomedical Sciences, University of Leeds, Leeds, UK; 5 National Genetic Centre, Royal Hospital, Muscat, Oman; 6 Institute of Medical Science, University of Toronto, Toronto, Ontario, Canada; 7 Department of Neuroscience, Centre for Addiction and Mental Health, Toronto, Ontario, Canada; 8 Institute of Cardiovascular and Medical Sciences, College of Medical, Veterinary and Life Sciences, University of Glasgow, Glasgow, UK; 9 Institute of Life Science, College of Medicine, Swansea University, Swansea, UK; 10 Department of Physiology, University of Toronto, Toronto, Ontario, Canada

## Abstract

Cognitive dysfunction is a core feature of dementia and a prominent feature in psychiatric disease. As non-redundant regulators of intracellular cAMP gradients, phosphodiesterases (PDE) mediate fundamental aspects of brain function relevant to learning, memory, and higher cognitive functions. Phosphodiesterase-4B (PDE4B) is an important phosphodiesterase in the hippocampal formation, is a major Disrupted in Schizophrenia 1 (DISC1) binding partner and is itself a risk gene for psychiatric illness. To define the effects of specific inhibition of the PDE4B subtype, we generated mice with a catalytic domain mutant form of PDE4B (Y358C) that has decreased ability to hydrolyze cAMP. Structural modeling predictions of decreased function and impaired binding with DISC1 were confirmed in cell assays. Phenotypic characterization of the PDE4B^Y358C^ mice revealed facilitated phosphorylation of CREB, decreased binding to DISC1, and upregulation of DISC1 and *β*-Arrestin in hippocampus and amygdala. In behavioral assays, PDE4B^Y358C^ mice displayed decreased anxiety and increased exploration, as well as cognitive enhancement across several tests of learning and memory, consistent with synaptic changes including enhanced long-term potentiation and impaired depotentiation *ex vivo.* PDE4B^Y358C^ mice also demonstrated enhanced neurogenesis. Contextual fear memory, though intact at 24 h, was decreased at 7 days in PDE4B^Y358C^ mice, an effect replicated pharmacologically with a non-selective PDE4 inhibitor, implicating cAMP signaling by PDE4B in a very late phase of consolidation. No effect of the PDE4B^Y358C^ mutation was observed in the prepulse inhibition and forced swim tests. Our data establish specific inhibition of PDE4B as a promising therapeutic approach for disorders of cognition and anxiety, and a putative target for pathological fear memory.

## INTRODUCTION

Cognitive dysfunction is a core feature of dementia and a prominent feature in major psychiatric disorders, such as mood and chronic psychotic disorders. Consequently, there is a large unmet need for cognition-enhancing drugs. The second messenger cyclic adenosine monophosphate (cAMP) mediates fundamental aspects of brain function relevant to learning, memory, and higher cognitive functions (Richter *et al*, 2013). Memory formation relies on expression of genes upregulated by the transcription factor CREB (cAMP response element binding protein), which is activated when phosphorylated by PKA (protein kinase A) downstream of cAMP. As a consequence, cAMP-specific phosphodiesterase enzymes, the sole regulators of cAMP gradients and ultimately CREB, are promising targets for the development of cognition-enhancing drugs (Ghavami *et al*, 2006; Richter *et al*, 2013).

The PDE4 family is cAMP-specific and comprises four subtypes (A–D). The expression patterns of individual PDE4 subtypes are clearly distinct at the regional and cellular level, suggesting that PDE4 subtypes serve non-redundant functions. Non-subtype-selective brain-penetrant PDE4 inhibitors (targeting all of four subtypes), such as rolipram, have shown therapeutic benefit in preclinical models of psychiatric and neurological diseases (Ghavami *et al*, 2006; Richter *et al*, 2013). These models include memory and cognition impairments induced by the *N*-methyl-D-aspartate receptor antagonist MK-801 (Davis and Gould, 2005; Zhang *et al*, 2000), cerebral ischemia-induced neuron loss and associated memory deficits in rats (Li *et al*, 2011a), age-related memory deficits (de Lima *et al*, 2008), and working, reference and associative memory deficits in a transgenic mouse model of Alzheimer’s disease (Gong *et al*, 2004).

However, non-selective PDE4 inhibitors are poorly tolerated in humans owing to nausea and emesis arising from inhibition of PDE4 in the brain stem (Mori *et al*, 2010) and gut (Menniti *et al*, 2006) at doses required for clinical effectiveness. Several lines of evidence suggest that these adverse effects are related to PDE4D, but not PDE4B (Robichaud *et al*, 2002). Indeed, despite comparable efficacy on other indicators, PDE4B-selective inhibitors may require doses approaching 100-fold that of PDE4D-selective agents to result in emesis, despite similar effect on other measures (Naganuma *et al*, 2009). Given the poor tolerability of non-selective agents, the elucidation of individual PDE4 subtype function has emerged as a strategy to guide the development of subtype-selective agents with maximal therapeutic utility and tolerability. This has, in part, been facilitated by the availability of knock-out (KO) mice deficient in individual PDE4 subtypes.

PDE4 subtypes are constitutively active enzymes containing a highly conserved catalytic domain, and then divided into categories defined by the presence of two unique, conserved domains: Upstream Conserved Region 1 (UCR1) and 2 (UCR2) (Zhang, 2009). The cAMP hydrolytic activity of PDE4B is facilitated and inhibited by PKA (Baillie, 2009) and ERK (extracellular signal-related kinase; Baillie *et al*, 2000), respectively. The catalytic domain and UCR1 contain phosphorylation sites for PKA and ERK, respectively. Five PDE4B isoforms have been identified in mammals: the long forms PDE4B1 (736 a.a.), 4B3 (721 a.a.) and 4B4 (659 a.a.), the short form 4B2 (564 a.a.), and the super-short form 4B5 (484 a.a.) (Cheung *et al*, 2007; Fatemi *et al*, 2008; Shepherd *et al*, 2003). The catalytic domain is common to all isoforms, whereas the long forms contain UCR1 and UCR2, the short form lacks UCR1, and the super-short form has only a portion of UCR2 (Zhang, 2009).

PDE4B is widely distributed throughout the brain in humans, monkeys, and rodents, with prominent expression in the cerebral cortex, limbic areas and diencephalon (Cherry and Davis, 1999; Lakics *et al*, 2010; Perez-Torres *et al*, 2000), as well as white matter tracts (Reyes-Irisarri *et al*, 2008). In cortex, the PDE4B1 isoform predominates, however in hippocampus and amygdala all isoforms are expressed (Reyes-Irisarri *et al*, 2008). Consistent with preclinical evidence of cognitive enhancement, changes in expression and subcellular localization of PDE4B in hippocampal neurons are associated with long-term potentiation (LTP) (Ahmed and Frey, 2005), considered one of the cellular mechanisms underlying learning and memory (Albensi *et al*, 2007). Moreover, *Pde4b* KO mice show an increase in the proliferation of neuronal cells in the hippocampal dentate gyrus (Zhang *et al*, 2008). Hippocampal slice preparations from *Pde4b* KO mice show markedly enhanced basal postsynaptic responses and long-term depression (Rutten *et al*, 2011).

*Pde4b* KO mice display a complex behavioral phenotype. They exhibit a moderately anxiogenic behavioral profile with decreased exploratory activity in the hole board and light-dark transition tests (Zhang *et al*, 2008), decreased locomotor activity in some open-field tests (Rutten *et al*, 2011; Siuciak *et al*, 2008; Zhang *et al*, 2008), and unaltered performance in the elevated plus maze (Siuciak *et al*, 2008). *Pde4b* KO mice perform normally in the fear conditioning (Rutten *et al*, 2011) and passive avoidance tests (Siuciak *et al*, 2008; Zhang *et al*, 2008), and show unaltered shock sensitivity (Rutten *et al*, 2011) and nociceptive responses (Siuciak *et al*, 2008; Zhang *et al*, 2008). In the Morris water maze, *Pde4b* KO mice show normal spatial memory acquisition and retention (Rutten *et al*, 2011; Siuciak *et al*, 2008; Zhang *et al*, 2008), but impaired reversal learning (Rutten *et al*, 2011). Acoustic startle response is increased in *Pde4b* KO mice, while prepulse inhibition of the startle response is decreased (Siuciak *et al*, 2008). They show decreased immobility in the forced swim test (Siuciak *et al*, 2008; Zhang *et al*, 2008), but not in the tail suspension test (Zhang *et al*, 2008). As expected, however, *Pde4b* KO mice show resistance to the inhibitory effects of rolipram on conditioned avoidance response (Siuciak *et al*, 2007). Though complex, this phenotype provides support for a role for PDE4B in both memory and anxiety.

Several lines of evidence have implicated PDE4B in major psychiatric illness, most notably schizophrenia. Disruption of PDE4B was identified by a chromosomal translocation in two first cousins with schizophrenia (Millar *et al*, 2005). Subsequently, large population genetic analyses of schizophrenia have inconsistently implicated single-nucleotide polymorphisms within PDE4B (Fatemi *et al*, 2008; Guan *et al*, 2012; Kahler *et al*, 2010; Numata *et al*, 2008; Pickard *et al*, 2007; Rastogi *et al*, 2009; Tomppo *et al*, 2009). Although an established rare genetic cause of schizophrenia, emerging primate data provide preliminary support for a role for PDE4B in the regulation of synaptic and spine plasticity in the dorsolateral prefrontal cortex and working memory (Paspalas *et al*, 2013). Thus, decreased PDE4B expression in post-mortem brains of patients with schizophrenia (Fatemi *et al*, 2008) may reflect compensatory downregulation of PDE4B to increase synaptic plasticity and counter the cognitive deficits associated with this condition, a possibility that has not received significant attention.

DISC1 is a large scaffolding protein that has important interactions with PDE4B (Millar *et al*, 2005; Murdoch *et al*, 2007). It plays key roles in neuronal development, and is a well-established risk factor for major mental illness associated with cognitive dysfunction (Blackwood *et al*, 2001; Porteous *et al*, 2014). There are five PDE4 binding sites on 100-kDa full-length DISC1; three of these sites are specific for PDE4B, while two potentially bind isoforms from each PDE4 subtype (Murdoch *et al*, 2007). In response to elevated cAMP levels, the shorter 71-kDa DISC1 isoform dissociates from PDE4B, whereas 100-kDa DISC1 does not dissociate, likely owing to more contact points with PDE4B (Murdoch *et al*, 2007).

Although poorly tolerated, non-subtype-selective PDE4 inhibitors have the potential to improve cognitive function, and several lines of evidence suggest that PDE4B may be a well-tolerated target for anxiety and cognitive enhancement. This study sought to determine the effects of specific inhibition of PDE4B by characterizing a catalytic domain mutant form of PDE4B (Y358C) that has decreased ability to hydrolyze cAMP. The catalytic domain of PDEs is an important pharmacological target, given limited homology between subtypes (Sung *et al*, 2003) and the well characterized relationship between existing PDE inhibitors and catalytic domains, notably clinically useful PDE5 inhibitors (Sung *et al*, 2003) and novel PDE4B inhibitors (Goto *et al*, 2013). We examined the neural and behavioral effects of the PDE4B-Y358C mutation in mice with a C57BL/6J genetic background. Our findings establish specific inhibition of PDE4B as a promising therapeutic approach for pathology affecting memory, anxiety, and fear memory.

## MATERIALS AND METHODS

### Generation of PDE4B Mutant

The catalytic domain of the PDE4B1 isoform (ENSMUSP00000102524) stretches from amino-acid residues (a.a.) 305–690 and is encoded by *Pde4b* exons 9–16 (Murdoch *et al*, 2007). We screened exon 10 (99 bp; a.a. 341–373) of *Pde4b* in 7776 male F_1_ progeny of ENU-mutagenized BALB/cAnN males and untreated C3H/HeH females in the MRC Harwell ENU DNA archive. In a single mouse (EMRCB/60.3d), we detected an adenine to guanine (A1073G) transition, corresponding to a Tyr^358^ (TAC)→Cys (TGC) (Y358C) exchange (Supplementary Figure 1a). The exon 10 sequences of the BALB/cAnN and C3H/HeH parental strains are identical, suggesting that the PDE4B^Y358C^ mutation arose as a result of ENU administration. The tyrosine at position 358 is present in PDE4B isoforms 1–5 (Supplementary Figure 1b) and is conserved across vertebrate species and in mouse PDE4A (Supplementary Figure 1c).

Heterozygous N_2_ backcross progeny of the founder PDE4B^Y358C/+^ (C3H/HeH × BALB/cAnN) F_1_ male and wild-type (WT) C57BL/6NTac females were backcrossed through the male and female lines to C57BL/6J for 10 generations before heterozygotes were intercrossed to generate homozygous mutant (PDE4B^Y358C/Y358C^) and WT (PDE4B^+/+^) littermates for phenotypic characterization. PDE4B^Y358C/+^ frozen embryos are available from the MRC Mammalian Genetics Unit, UK (har.mrc.ac.uk).

Full methods are available in the Supplementary Methods.

Sex-differences were explored with two-way analysis of variance (ANOVA), however, no significant Genotype × Sex interactions were observed. For parsimonious interpretation, statistical differences are reported using Student’s *t*-test, linear regression, repeated measures ANOVA, and Cox regression. *Post hoc* tests were performed using least significant difference when significant genotype*test interactions emerged in ANOVA or repeated measures ANOVA.

## RESULTS

**Figure 1:**
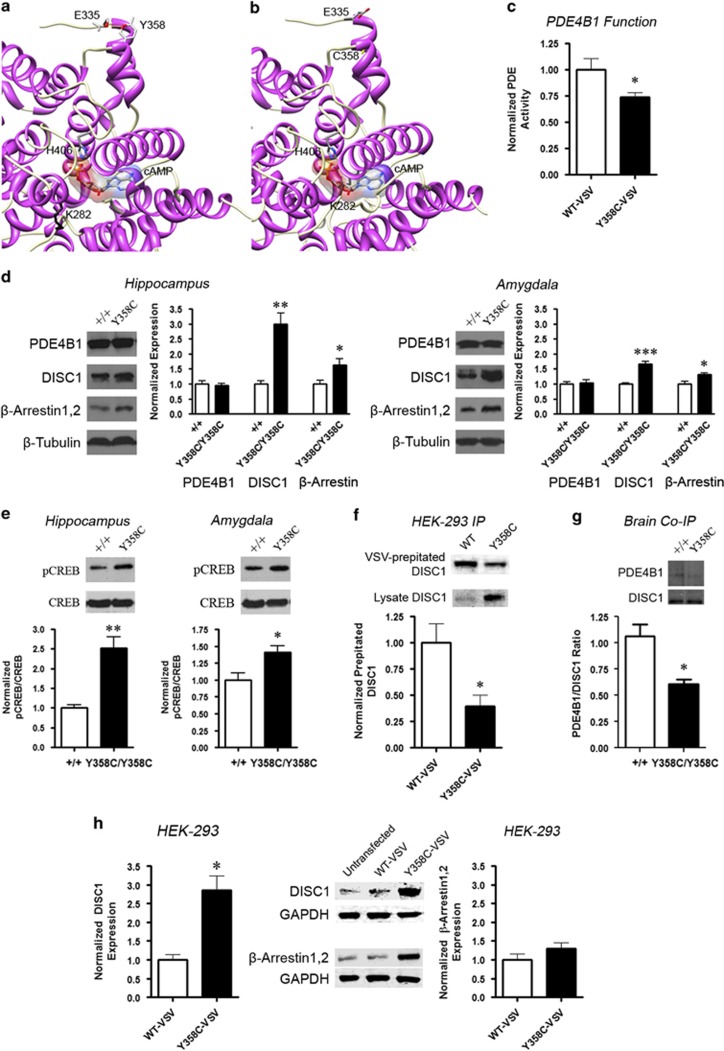
Biochemical consequences of PDE4B-Y358C mutation. (a) Wild-type PDE4B1 with accessible Y358 and H406 interacting with cAMP. (b) Mutant PDE4B1 with inaccessible 358C and disrupted cAMP-docking position. (c) cAMP hydrolytic function of VSV-epitope-tagged PDE4B1-Y358C and WT constructs expressed in HEK-293 cells (paired-*t*(5)=2.90, *p*<0.05). (d) No PDE4B1 expression differences in hippocampus or amygdala. PDE4B^Y358C/Y358C^ mice have increased expression of DISC1 and *β*-arrestin1,2 specific to the hippocampus (*β*-Arrestin1,2 *t*(6)=2.66, *p*<0.05; DISC1 *t*(6)=5.06, *p*<0.01) and amygdala (*β*-Arrestin1,2 *t*(14)=2.94, *p*<0.05; DISC1 *t*(6)=6.56, *p*<0.001). (e) Increased phosphorylation of CREB in the PDE4B^Y358C/Y358C^ hippocampus (*t*(6)=5.17, *p*<0.01) and amygdala (*t*(6)=2.89, *p*<0.05). (f) Lower DISC1 immunoprecipitation in HEK-293 cells expressing VSV-epitope-tagged Y358C PDE4B1 (*t*(4)=2.90, *p*<0.05). (g) Whole brain co-immunoprecipitation demonstrating impaired PDE4B1–DISC1 binding in PDE4B^Y358C/Y358C^ mice (*t*(9)=3.74, *p*<0.01). (h) Increased DISC1 (*t*(4)=4.48, *p*<0.05) but not *β*-Arrestin1,2 (*t*(4)=1.39, NS) expression in HEK-293 cells expressing VSV-epitope-tagged PDE4B1–Y358C constructs. Means±SEM in all graphs, *p<0.05, **p<0.01, ***p<0.001. cAMP, cyclic adenosine monophosphate; DISC1, Disrupted in Schizophrenia 1; NS, not significant.

At the cAMP binding site, there is an interaction between the central phosphate group of cAMP and H406 in WT PDE4B1 (Figure 1a). Though the Y358 residue is located within the catalytic domain, it is neither at the site of cAMP binding nor rolipram binding (Richter *et al*, 2001). In the Y358C variant, a conformational change is predicted to the binding site by introducing a beta conformational bend bordering the cAMP binding cavity around K282. This severely disrupts the docking position of cAMP (Figure 1b) as the side chain of K282 bisects the binding site.

### PDE4B Y358C Alters cAMP Signaling and CREB Phosphorylation

Using VSV-epitope-tagged human PDE4B1-Y358C and WT constructs expressed in HEK-293 cells, we found that PDE4B1-Y358C has a 27% decreased ability to hydrolyze cAMP (Figure 1c). In mouse hippocampus, RT-PCR of PDE4B1–5 did not detect expression differences between PDE4B^Y358C/Y358C^ and PDE4B^+/+^ mice (Supplementary Figure 2a). Similarly, western blotting did not detect genotypic differences in expression of PDE4B1 in the hippocampus, amygdala, prefrontal cortex, and nucleus accumbens associated with Y358C (Figure 1d; Supplementary Figure 2b and c). We probed the expression of PDE4A5, due to Y358 conservation in mouse, and PDE4D3, due to signs of upregulation in *Pde4b* KO mice (Zhang *et al*, 2008), but found no genotypic differences (Supplementary Figure 2d). Hippocampal slices from PDE4B^Y358C/Y358C^ brains have similar levels of cAMP as PDE4B^+/+^, but showed a greater cAMP accumulation when challenged with forskolin alone or in combination with rolipram (Supplementary Figure 2e). As PDE4B regulates cAMP gradients and ultimately CREB, we examined total expression of CREB and its phosphorylation (pCREB), finding increased pCREB/CREB in the hippocampi (2.5-fold) and amygdala (1.4-fold) of PDE4B^Y358C/Y358C^ mice (Figure 1e). The Y358C variant of PDE4B is thus normally expressed, but has reduced enzymatic activity, which in turn primes CREB signaling.

### Y358C Affects the PDE4B Partners DISC1 and *β*-Arrestin

As Y358 occurs within one of the three DISC1 binding sites on PDE4B1 (a.a. 352–380) (Murdoch *et al*, 2007), we probed 100-kDa DISC1 binding. Expression of the VSV-epitope-tagged PDE4B1-Y358C and WT constructs in HEK-293 cells revealed decreased DISC1 immunoprecipitation (Figure 1f), which was paralleled in co-immunoprecipitation from PDE4B^Y358C/Y358C^ brains (Figure 1g). Western blotting revealed that the expression of DISC1 was unaltered in the prefrontal cortex and nucleus accumbens (Supplementary Figure 1c and d), but was increased 3-fold in the hippocampus and 1.6-fold in the amygdala of PDE4B^Y358C/Y358C^ mice (Figure 1d). The DISC1 upregulation was confirmed using VSV-epitope-tagged human PDE4B1-Y358C and WT constructs expressed in HEK-293 cells (Figure 1h). *β*-Arrestins are known to recruit PDE4 to the *β*2-adrenoreceptor, thus controlling PKA activity at the membrane (Baillie *et al*, 2003; Li *et al*, 2011b). Though PDE4B1-*β*-Arrestin1/2 binding was not impaired in PDE4B^Y358C/Y358C^ mice (Supplementary Figure 2f), *β*-Arrestin1/2 was increased 1.6-fold in the hippocampus and 1.3-fold in the amygdala (Figure 1d), but not in prefrontal cortex and nucleus accumbens (Supplementary Figure 2b and c).

### PDE4B Y358C Mice Display Decreased Anxiety and Greater Exploratory Behavior

**Figure 2:**
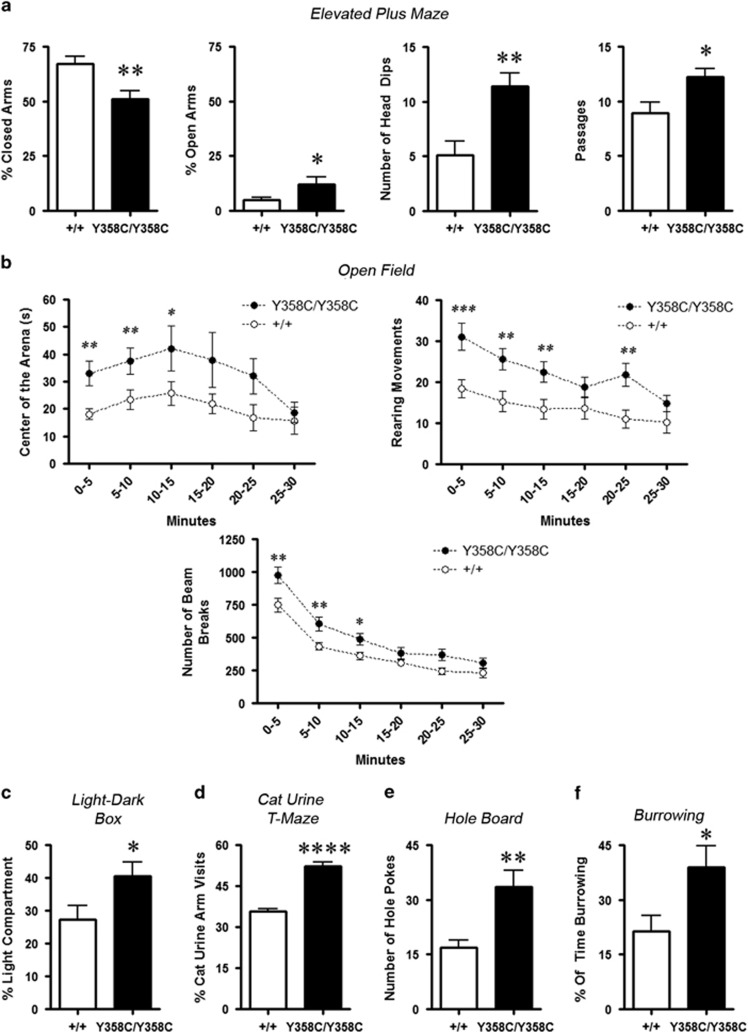
Anxiety and exploration. (a) Elevated plus maze. PDE4B^Y358C/Y358C^ mice (*n*=9M/6F) spent less time than PDE4B^+/+^ (*n*=10M/5F) in the closed arms (*t*(28)=3.28, *p*<0.01), more time in the open arms (*t*(28)=2.09, *p*<0.05), performed more head dips (*t*(28)=3.66, *p*<0.01) and more passages between arms (*t*(28)=2.59, *p*<0.05). (b) Open-field. PDE4B^358C/358C^ mice (*n*=13M/8F) spent more time than PDE4B^+/+^ (*n*=12M/9F) in the centre of the arena (Time F(5, 200)=4.43, *p*<0.001; Genotype F(1, 200)=5.01, *p*<0.05; Time × Genotype F(1, 200)=0.84, NS), more rearing movements (Time F(5, 200)=21.83, *p*<0.0001; Genotype F(1, 200)=7.30, *p*<0.01; Time × Genotype F(1, 200)=3.28, *p*<0.01), and more total beam breaks (Genotype: F(1, 200)=8.06, *p*<0.01); Time: F(5, 200)=127.1, p≤0.0001; Genotype × Time: F(5, 200)=2.32, *p*<0.05). (c) Light–dark box. PDE4B^Y358C/Y358C^ (*n*=6M/5F) mice spent more time than PDE4B^+/+^ (*n*=7M/5F) in the light compartment (t(21)=2.16, *p*<0.05). (d) Aversion to cat odor. PDE4B^+/+^ (*n*=5M/3F) mice avoided the bobcat urine baited arm whereas PDE4B^358C/358C^ (*n*=5M/5F) visited the both arms equally (*t*(16)=8.71, *p*<0.0001). (e) Holeboard. PDE4B^Y358C/Y358C^ mice (*n*=6M/1F) performed more hole pokes than controls (*n*=6M/2F); t(13)=3.54, *p*<0.01). (f) Food burrowing. PDE4B^Y358C/Y358C^ mice (*n*=5M/5F) spent significantly more time foraging than PDE4B^+/+^ (*n*=5M/5F; *t*(18)=2.42, *p*<0.05). Means±SEM in all graphs, *p<0.05, **p<0.01, ***p<0.001. F, female; M, male; NS, not significant.

In the elevated plus maze, mice face a conflict between aversion to open arms and motivation to explore these arms. PDE4B^Y358C/Y358C^ mice spent more time in the open arms and made more exploratory head dips and passages than PDE4B^+/+^ mice (Figure 2a). In a novel open field, PDE4B^Y358C/Y358C^ mice displayed greater ambulation and rearing activity, and spent more time in the aversive center of the arena (Figure 2b). Further, PDE4B^Y358C/Y358C^ mice spent more time in the bright compartment of the light–dark box (Figure 2c). We exploited murine aversion to cat odors (Vyas *et al*, 2007) by baiting a T-maze with food pellets in one arm and bobcat urine in the opposite arm. PDE4B^+/+^ mice avoided the bobcat urine arm, whereas PDE4B^Y358C/Y358C^ mice explored both arms equally (Figure 2d). This difference was not attributable to impaired olfaction (Supplementary Figure 3a).

In a holeboard test of exploratory behavior, PDE4B^Y358C/Y358C^ mice performed more nose-pokes than PDE4B^+/+^ mice (Figure 2e). When attempting to find buried food, PDE4B^Y358C/Y358C^ mice engaged in greater exploratory burrowing than PDE4B^+/+^ mice (Figure 2f). This was not merely hyperlocomotion (Supplementary Figure 3b). PDE4B^Y358C/Y358C^ mice thus exhibited a consistent pattern of lower anxiety, and greater exploratory behavior and risk-taking.

We did not observe differences in depressive-like behavior using the forced swim test (Supplementary Figure 3b).

### PDE4B Y358C Mice Display Enhanced Learning and Memory

**Figure 3:**
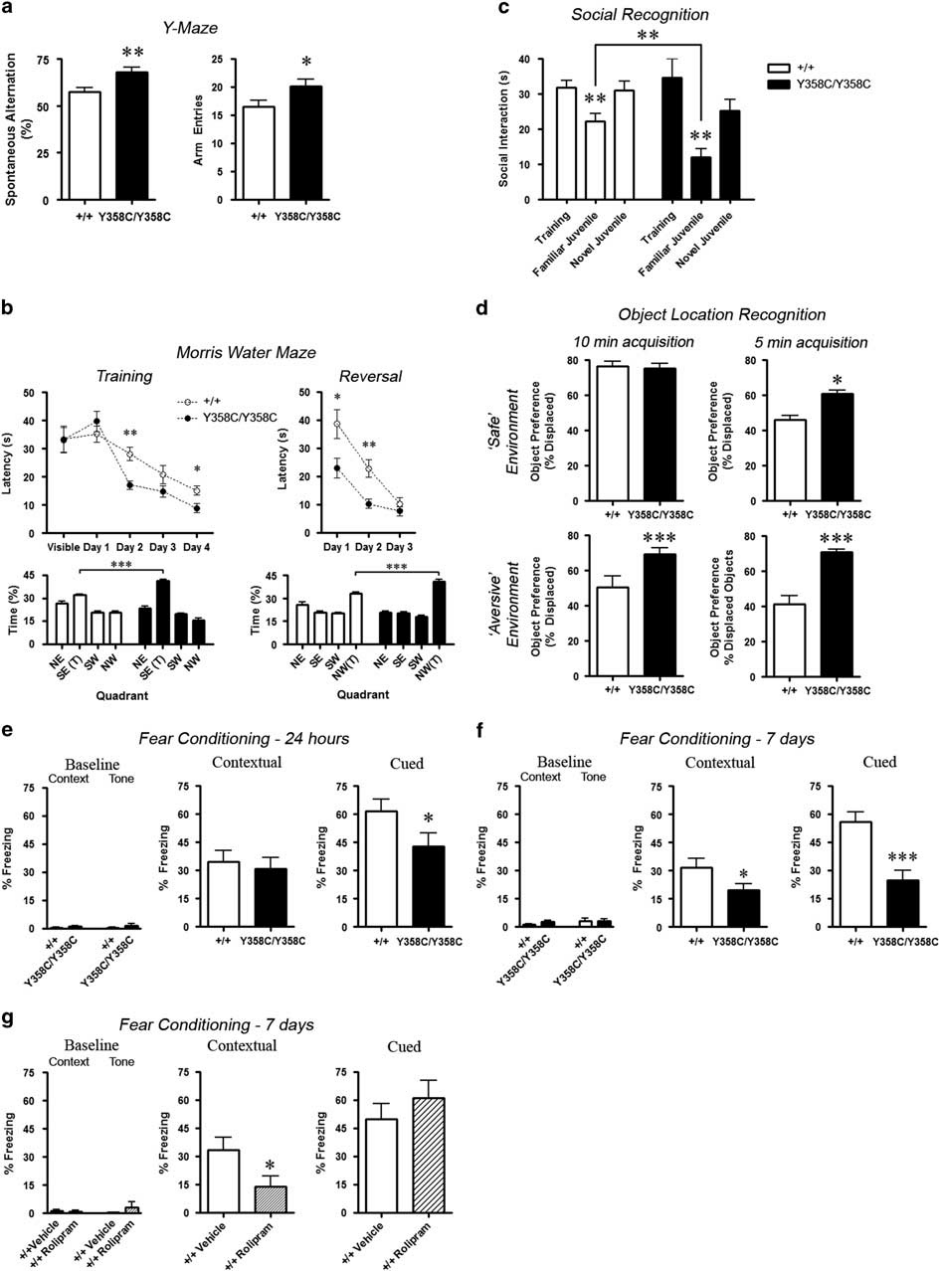
Cognitive enhancement and fear memory. (a) Y-maze. PDE4B^Y358C/Y358C^ mice (*n*=11M/11F) demonstrated improved working memory with more spontaneous alternation (*n*=10M/17F; *t*(47)=3.02, *p*<0.01) and increased arm entry (*t*(47)=2.27, *p*<0.05) than PDE4B^+/+^. (b) Morris water maze. PDE4B^Y358C/Y358C^ mice (*n*=4M/4F) demonstrated more rapid spatial memory acquisition than PDE4B^+/+^ (*n*=6M/3F) during the training (Genotype F(1,45)=2.27, *p*=0.059); Genotype × Time F(3,45)=4.61, *p*<0.01) and reversal learning (Genotype F(1,30)=8.80, *p*<0.01; Genotype × Day F(2,30)=3.73, *p*<0.05) phases. PDE4B^Y358C/Y358C^ mice spent more time in the target quadrant on the probe trial after training (Quadrant F(3, 60)=133.6, *p*<0.0001; Genotype × Quadrant F(3,60)=20.31, *p*<0.0001) and after reversal learning (Quadrant F(3, 32)=110.6, *p*<0.0001; Genotype × Quadrant F(3,32)=21.22, *p*<0.0001). (c) Social recognition. PDE4B^Y358C/Y358C^ mice exhibit improved social memory (Y358C *n*=7M *vs* WT *n*=9M; *t*(14)=3.13, *p*<0.01). (d) Displaced object location recognition. Ten-minute acquisition in ‘safe’ environment (top left) reveals comparable displaced object preference between genotypes. Five-minute acquisition in a ‘safe’ environment (top right; Y358C 10M/2F *vs* WT 10M/2F; *t*(22)=4.58, *p*<0.0001), 5-min acquisition in a ‘aversive’ environment (bottom right; *t*(13)=6.64, *p*<0.0001), and 10-min acquisition in an ‘aversive’ environment (bottom left; *t*(14)=2.60, *p*<0.05) all reveal greater displaced object preference in PDE4B^Y358C/Y358C^ mice. (e) Fear conditioning—24 h. PDE4B^Y358C/Y358C^ mice (*n*=7M/5F) exhibit normal contextual and decreased cued freezing compared to PDE4B^+/+^ (*n*=6M/6F) 24 h after fear conditioning (*t*(22)=1.99, *p*<0.05). (f) Fear conditioning—7 days. PDE4B^Y358C/Y358C^ (*n*=7M/6F) mice exhibit decreased contextual (*t*(25)=2.08, *p*<0.05) and decreased cued (*t*(25)=4.08, *p*<0.001) freezing compared to PDE4B^+/+^ (*n*=10M/4F) 7 days after fear conditioning. (g) Fear conditioning—rolipram. Subchronic rolipram (1 mg/kg twice daily) reproduced decreased contextual freezing 7 days after training in +/+ mice (rolipram *n*=8M/1F *vs* vehicle *n*=7M/1F; *t*(15)=2.27, *p*<0.05). Means±SEM in all graphs, *p<0.05, **p<0.01, ***p<0.001. F, female; M, male; NS, not significant; WT, wild type.

In the Y-maze spontaneous alternation test, PDE4B^Y358C/Y358C^ mice exhibited improvements in working spatial memory (Figure 3a). In the Morris water maze, PDE4B^Y358C/Y358C^ mice located the escape platform faster than PDE4B^+/+^ mice in both acquisition and reversal training trials (Figure 3b), an effect not attributable to swimming time or speed (Supplementary Figure 3b and c). Moreover, PDE4B^Y358C/Y358C^ mice had improved performance compared to PDE4B^+/+^ mice in probe trials 24 h after the last acquisition and reversal trials (Figure 3b). In a social recognition test, PDE4B^Y358C/Y358C^ mice demonstrated enhanced long-term (24 h) memory of a familiar juvenile compared with PDE4B^+/+^ mice (Figure 3c).

Object location recognition is a hippocampus-dependent task exploiting the natural exploratory activity of rodents toward spatial novelty to assess the detection of spatial relocation of a known object (Stupien *et al*, 2003). PDE4B^Y358C/Y358C^ and PDE4B^+/+^ mice displayed similar preferences for displaced objects following a 10-min acquisition period, but only PDE4B^Y358C/Y358C^ mice demonstrated a preference for displaced objects when the acquisition period was reduced to 5 min (Figure 3d). We have previously shown that decreasing environmental threat by dimming the lights results in increased exploration with consequent improvement in memory (Saab *et al*, 2009). As PDE4B^Y358C/Y358C^ mice already display increased exploratory behavior under typical room lighting (‘bright lights’ Figure 2), we sought to increase the environmental threat by exposing mice to brighter lights and a transparent arena floor at 1-m elevation. In this more aversive environment, PDE4B^+/+^ mice failed to show preference for displaced objects following 10 min of acquisition, but the displaced object preference of PDE4B^Y358C/Y358C^ mice was maintained, even when acquisition was limited to 5 min (Figure 3d).

### PDE4B Y358C Mice Display Altered Fear Memory

In the fear conditioning paradigm, PDE4B^Y358C/Y358C^ mice demonstrated levels of freezing comparable with PDE4B^+/+^ mice in the hippocampus-dependent contextual memory test 24 h after conditioning, but showed decreased freezing in the amygdala-dependent cued memory test (Figure 3e). When a portion of this cohort was retested 7 days after conditioning, PDE4B^Y358C/Y358C^ mice displayed less freezing to the context than PDE4B^+/+^ mice (Supplementary Figure 4a). Therefore, 7-day fear memory was tested in an independent cohort, in which PDE4B^Y358C/Y358C^ mice showed lower levels of both contextual freezing and cued freezing after 7 days, in the absence of exposure at 24 h (Figure 3f). The PDE4B^Y358C/Y358C^ decreased freezing is not attributable to altered nociception, or to sensorimotor processing as assessed using the prepulse inhibition test (Supplementary Figure 4b–d).

To further examine the effect of PDE4B functional impairment on 7-day fear memory, twice daily injections of the non-selective PDE4 inhibitor rolipram (1 mg/kg) were administered to PDE4B^+/+^ mice from 24 h to 6 days after conditioning. Compared with vehicle-treated controls, the rolipram-treated mice exhibited a contextual memory-specific reduction in freezing (Figure 3g), supporting our PDE4B^Y358C/Y358C^ findings.

### PDE4B Y358C Mice Display Altered Synaptic Plasticity

**Figure 4:**
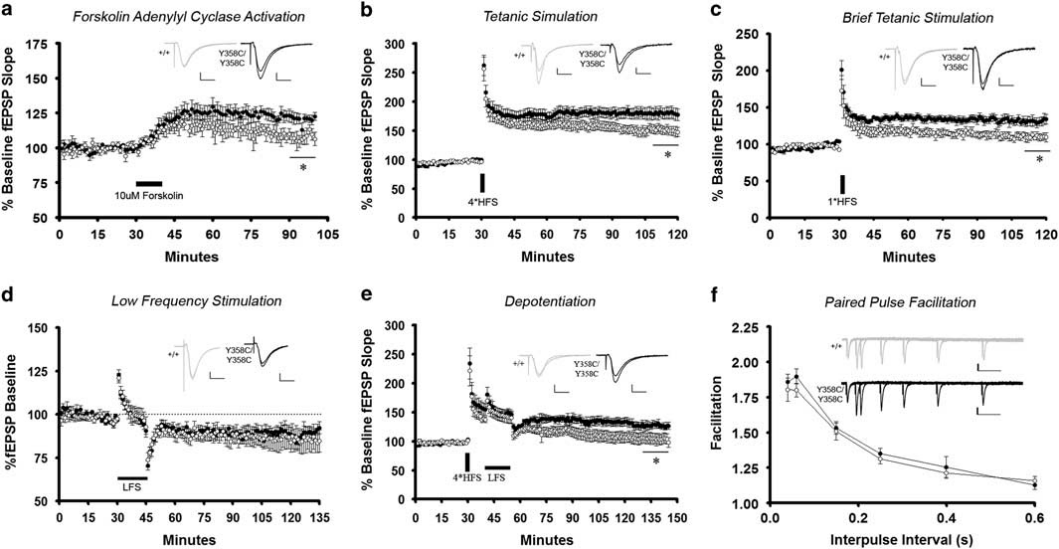
Synaptic plasticity in Schaffer collaterals of hippocampal CA1 neurons. (a) Enhanced potentiation after forskolin challenge in PDE4B^Y358C/Y358C^ slices (*t*(7)=2.62, *p*<0.05; PDE4B^Y358C/Y358C^ four slices from three mice; PDE4B^+/+^ five slices from three mice). (b) Facilitated and stable LTP upon tetanic stimulation in PDE4B^Y358C/Y358C^ slices (*t*(18)=2.36, *p*<0.05; PDE4B^Y358C/Y358C^ 11 slices from 10 mice, PDE4B^+/+^ 10 slices from 6 mice). (c) Facilitated and stable LTP from brief tetanic stimulation in PDE4B^Y358C/Y358C^ slices (*t*(10)=2.83, *p*<0.05; PDE4B^Y358C/Y358C^ six slices from five mice, PDE4B^+/+^ six slices from six mice). (d) Low-frequency stimulation results in comparable LTD (PDE4B^Y358C/Y358C^ six slices from three mice, PDE4B^+/+^ six slices from three mice). (e) Impaired depotentiation in PDE4B^Y358C/Y358C^ mice (PDE4B^Y358C/Y358C^ seven slices from seven mice, PDE4B^+/+^ nine slices from nine mice). (f) Y358C mutation does not affect pre-synaptic short-term plasticity. Representative fEPSPs are presented for each experiment (PDE4B^+/+^ gray, PDE4B^Y358C/Y358C^ black). For panels a–d the scale represents 10 ms and 0.2 mV, and 100 ms and 0.2 mV in panel f. Means±SEM in all graphs, *p<0.05. LTP, long-term potentiation.

Hippocampal CA1 electrophysiological experiments were used to explore synaptic plasticity in PDE4B^Y358C/Y358C^ mice. The Y358C mutation did not affect basal synaptic transmission (Supplementary Figure 5). We applied forskolin, an adenylyl cyclase activator, and found increased potentiation in PDE4B^Y358C/Y358C^ hippocampal slices, confirming decreased PDE4B–Y358C cAMP hydrolytic function (Figure 4a). To examine the effect of sustained electrical stimulation on LTP in PDE4B^Y358C/Y358C^ mice, we utilized high frequency (100-Hz) trains and varied their number. Following tetanic stimulation with four trains, PDE4B^Y358C/Y358C^ slices demonstrated enhanced potentiation (Figure 4b). Given the rapid acquisition observed in object recognition (Figure 3d), we employed a single 100-Hz train, which is below the typical threshold for LTP (Albensi *et al*, 2007). PDE4B^Y358C/Y358C^ slices showed evidence of facilitation, whereas PDE4B^+/+^ slices, as expected, demonstrated non-significant potentiation (Figure 4c). We examined synaptic depression in PDE4B^Y358C/Y358C^ mice, but observed no change in slices from 16–17-day-old mice after 900 pulses of 1-Hz stimulation (Figure 4d). As our behavioral studies were conducted on 8–12 week old mice, we also studied an adult form of synaptic depression—depotentiation—whereby tetanic stimulation is followed by low frequency stimulation (Grunwald *et al*, 2001). Depotentiation restored fEPSP slopes to baseline in PDE4B^+/+^ slices but resulted in less synaptic depression in PDE4B^Y358C/Y358C^ slices (Figure 4e). Paired-pulse facilitation showed no evidence that Y358C impaired presynaptic short-term plasticity (Figure 4f).

### PDE4B Y358C Mice Display Increased Dendritic Spine Density and Hippocampal Neurogenesis

**Figure 5:**
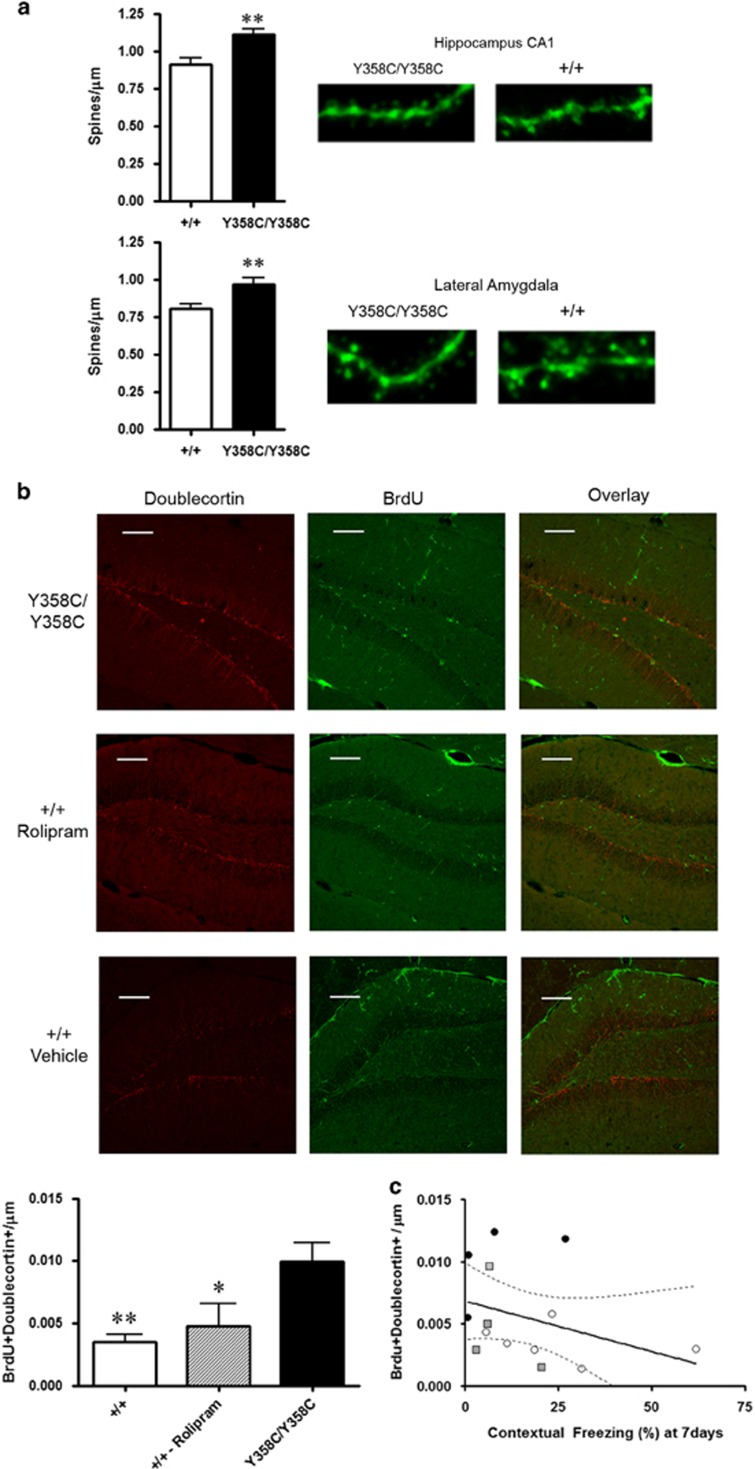
Dendritic spine density and neurogenesis. (a) Increased dendritic spine density in both the hippocampus (34 segments from 4 *Thy1*-GFP mice; *t*(32)=3.57, *p*<0.01) and the amygdala (43 segments from 4 *Thy1*-GFP mice; *t*(41)=3.01, *p*<0.01) of PDE4B^Y358C/Y358C^ mice. (b) Increased dentate neurogenesis among PDE4B^Y358C/Y358C^ mice compared with control mice receiving rolipram 1 mg/kg or vehicle twice daily for 6 days (F(2,12)=8.80, *p*<0.01). (c) Subgranual layer neurogenesis was not related to contextual freezing 7 days after fear conditioning (F(1, 13)=2.01, NS). *p<0.05, **p<0.01. GFP, green fluorescent protein; NS, not significant.

The combined administration of rolipram and antidepressants to rodents results in increased BDNF expression (Fujimaki *et al*, 2000) and CA1 spine density (Marchetti *et al*, 2010). In mouse models of Alzheimer’s disease, rolipram restores dendritic spine density (Smith *et al*, 2009), while *Disc1* mutant mice with impaired DISC1-PDE4B binding show alterations in hippocampal spine density (Lee *et al*, 2011). We therefore sought to examine dendritic spine density in PDE4B^Y358C/Y358C^ mice, focusing on the hippocampus and lateral amygdala. Using the *Thy1*-GFP transgene (Feng *et al*, 2000) as a reporter for dendritic spines, we identified greater spine densities in both the hippocampus and lateral amygdala of PDE4B^Y358C/Y358C^ mice (Figure 5a).

Enhanced adult hippocampal neurogenesis has been observed in both *Pde4b* and *Pde4d* KO mice (Li *et al*, 2011b; Zhang *et al*, 2008). In light of data indicating that adult hippocampal dentate neurogenesis destabilizes contextual fear memory (Akers *et al*, 2014), we probed neurogenesis in conjunction with fear conditioning. Using daily injections of 5-bromo-2′-deoxyuridine (BrdU; 50 mg/kg, i.p.) for four days following fear conditioning, we examined neurogenesis in the hippocampal dentate gyrus of PDE4B^Y358C/Y358C^ mice in comparison with PDE4B^+/+^ mice that received rolipram (1 mg/kg) or vehicle twice daily for 6 days. Increased numbers of dentate BrdU+ and doublecortin+ cells were observed in PDE4B^Y358C/Y358C^ mice compared with both rolipram-treated and vehicle-treated PDE4B^+/+^ mice (Figure 5b). However, no significant relationship was observed between adult hippocampal neurogenesis and contextual freezing with linear regression (Figure 5c).

## DISCUSSION

The present study sought to determine the neural and behavioral effects of a catalytic domain mutant form of PDE4B (Y358C) that has decreased ability to hydrolyze cAMP. Consistent with previous data suggesting the involvement of the Y358 residue in the interaction of PDE4B with DISC1 (Murdoch *et al*, 2007), our comparative molecular modeling suggested that the cysteine substitution resulted in a conformational modification rendering the DISC1 interaction site inaccessible. Confirmed in cell culture and brain tissue, the decreased binding of PDE4B-Y358C to DISC1 was associated with increased expression of DISC1 and *β*-arrestin1/2 in the amygdala and hippocampus, perhaps indicating compensatory mechanisms to normalize PDE4B activity. Moreover, our modeling suggested impaired cAMP binding owing to tertiary changes as a result of the Y358C substitution. Indeed, 27% impairment in cAMP hydrolytic ability of PDE4B1-Y358C observed *in vitro* is proportional to that of physiological regulation by phosphorylated ERK (Baillie *et al*, 2000). The importance of the Y358C alteration was confirmed *ex vivo* in forskolin-challenged hippocampal slices, which demonstrated rapid cAMP accumulation and sustained potentiation at Schaffer CA1 collaterals.

PDE4B^Y358C/Y358C^ mice consistently demonstrated low levels of anxiety in several tests, and even failed to demonstrate the natural robust innate fear response to cat odor. A decreased fear response to cat odor is also shown by mice infected with *Toxoplasma gondii* (Vyas *et al*, 2007), a schizophrenia risk factor that localizes to the lateral amygdala involved in both innate and learned fear (LeDoux, 2000). *Pde4b* KO mice show anxiogenic-like behaviour in the holeboard and light-dark transition tests (Zhang *et al*, 2008), and therefore null mutation (KO) and missense mutation (Y358C) of *Pde4b* appear to have opposite effects on some tests of anxiety (Rutten *et al*, 2011; Siuciak *et al*, 2008; Zhang *et al*, 2008). Such phenotypic differences between mice which harbor a missense mutation or null mutation (KO) of the same gene are not uncommon; for example, missense mutation I810N (Kirshenbaum *et al*, 2013) and KO (Ikeda *et al*, 2013) alleles of the Na^+^,K^+^–ATPase α3 gene are reported to have opposite effects on the beam walking assay. Our finding that reduced-function of PDE4B by a catalytic domain mutation results in anxiolytic effects is consistent with anxiolysis observed with non-selective PDE4 inhibitors in rodents (Li *et al*, 2009b; Silvestre *et al*, 1999) and primates (Rutter *et al*, 2014). Altogether these data suggest that the anxiolytic effects of non-selective PDE4 inhibitors may be PDE4B dependent.

In humans and mice, exploratory tendencies are predictive of general cognitive abilities (Matzel *et al*, 2006). However, an increased exploratory tendency does not equate to improvement in general cognitive performance (Light *et al*, 2008, 2011), perhaps suggesting a common substrate yet lack of causality between these factors. Reducing an environment’s aversive characteristics can shift the motivation underlying exploration, resulting in learning facilitation (Saab *et al*, 2009). The PDE4B-Y358C mutation decreased fear responses and increased exploration in mice, and we observed a consistent pattern of cognitive enhancement in PDE4B^Y358C/Y358C^ mice in non-aversive tests. The resistance shown by PDE4B^Y358C/Y358C^ mice to the negative influence of environmental threat on object location memory formation suggests dissociation between fear and memory formation. Our learning and memory as well as synaptic findings are consistent with the PDE4B–Y358C mutant’s reduced cAMP hydrolytic activity and facilitation of CREB phosphorylation (Tully *et al*, 2003).

In fear conditioning, inputs are received within the lateral amygdala to form an association between the auditory tone (conditioned stimulus) and the foot-shock (unconditioned stimulus) (LeDoux, 2000). The deficits in PDE4B^Y358C/Y358C^ cued memory may reflect altered lateral amygdala function, consistent with *β*-Arrestin upregulation and the *β*-Arrestin–PDE4 complex required for fear conditioning (Li *et al*, 2009a). Yet PDE4B^Y358C/Y358C^ mice had intact contextual (hippocampal) fear memory at 24 h, which is considered sufficient time for the formation of long-term memories (Tully *et al*, 2003). *Pde4b* KO mice, by contrast, have shown no differences in context-dependent and cue-dependent fear memory tests at 24 h (Rutten *et al*, 2011). The decrease in contextual freezing exhibited by PDE4B^Y358C/Y358C^ mice when tested at 7 days is unlikely to represent extinction of fear memory, as lower contextual freezing after 7 days was observed independent of pre-exposure to the context at 24 h. Our data suggest that this is due to PDE4B dysfunction rather than disrupted interaction with DISC1 because the PDE4B^Y358C/Y358C^ fear conditioning phenotype was replicated in control mice given subchronic rolipram, which inhibits PDE4B activity but does not affect binding to DISC1. Moreover, the replication of the phenotype when rolipram was initiated 24 h after fear conditioning suggests that PDE4B is involved in a very late process required for long-term memory persistence.

Impaired regulation of cAMP signaling in the hippocampus by PDE4B may impair a very late-phase of consolidation, perhaps by poor coordination of the late-phase protein transcription required for long-term memory persistence. Disruption of late-phase processes by injecting anisomycin or BDNF-antibodies into CA1 of the hippocampus 12 h after fear conditioning leads to a similar phenotype, with intact freezing at 48 h but decreased freezing after 7 days (Bekinschtein *et al*, 2007). Moreover, this phenotype has also been reported with antidepressants given 12 h after fear conditioning (Slipczuk *et al*, 2013). However, unlike the PDE4B^Y358C/Y358C^ phenotype, these previous studies detected no change in fear memory at 7 days if the interventions occurred at or after 24 h (Bekinschtein *et al*, 2007; Slipczuk *et al*, 2013).

Alternatively, the degree to which the PDE4B–Y358C function facilitates the acquisition of new associations and formation of new synapses may lead to loss of behavioral specificity over time. Moreover, neurogenesis in PDE4B^Y358C/Y358C^ mice may destabilize the fear trace (Akers *et al*, 2014), yet in our mice this was not linearly related to contextual fear memory. Our findings suggest that PDE4B inhibition is a putative therapeutic approach in overly persistent fear memories, typified by post-traumatic stress disorder, which would benefit from a larger prophylactic window.

In summary, the Y358C reduced-function PDE4B mutant resulted in increased phosphorylation of CREB, decreased binding of PDE4B to DISC1, and upregulation of DISC1 and *β*-Arrestin in the hippocampus and amygdala. PDE4B^Y358C/Y358C^ mice displayed a phenotype of decreased anxiety, increased exploration, and cognitive enhancement across several tests of learning and memory, in parallel with hippocampal synaptic changes including enhanced LTP, impaired depotentiation, and enhanced neurogenesis. Contextual fear memory, though intact at 24 h, was decreased at 7 days and replicated pharmacologically with a non-selective PDE4 inhibitor. Our data establish specific inhibition of PDE4B as a promising therapeutic approach for disorders of memory and anxiety. Future studies should examine the neural and behavioral effects of brain-penetrant PDE4B-selective inhibitors in psychiatric and neurologic models.

## FUNDING AND DISCLOSURE

This work was supported in part by grants from the Canadian Institutes of Health Research (MOP-111198; to JCR), the United Kingdom Medical Research Council (G0900625) and the National Alliance for Research on Schizophrenia and Depression (to SJC). JCR holds a Canada Research Chair in Learning and Memory. The remaining authors declare no conflict of interests.

## Supplementary Material

Supplementary Figure Legneds
