# Supplementary material for: Specific Inhibition of Phosphodiesterase-4B Results in Anxiolysis and Facilitates Memory Acquisition
Source: Neuropsychopharmacology. 2015 Sep 2;41(4):1080–92. doi: 10.1038/npp.2015.240 (PMC4748432; doi:10.1038/npp.2015.240)
Supplement: Supplementary Figure Legneds [file npp2015240x1.doc]

**Supplementary Figure 1.** (**a**) Sequencing chromatogram demonstrated an A1073G transition in a conserved region resulting in the Y358C missense mutation. (**b**) PDE4B isoform alignment (blue – UCR2; red – catalytic domain; gray – conserved amino acid residues; yellow – Y358C position). (**c**) Y358 is conserved across species and in PDE4A.

**Supplementary Figure 2.** (**a**) Real-Time PCR analysis of hippocampal PDE4B isoforms (PDE4B^Y358C/Y358C^ n=4, PDE4B^+/+^ n=4, F(1,4)=0.20, *ns*). (**b-c**) Representative western blots for PDE4B1, DISC1, and β-Arrestin1,2 for the prefrontal cortex and nucleus accumbens. (**d**) Expression of PDE4A5 and PDE4D3 in hippocampus. (**e**) cAMP quantification in hippocampal slices. (**f**) Representative co-IP for PDE4B1-β-Arrestin1,2.

**Supplementary Figure 3.** (**a**) PDE4B^Y358C/Y358C^ mice do not demonstrate impaired olfactory function (t(18)=1.23, ns). (**b**) PDE4B^Y358C/Y358C^ (n=6M/5F) mice do not demonstrate changes compared to PDE4B^+/+^ mice (n=8M/10F) in depressive behaviors or hyperlocomotion in the forced swim test (t(27)=0.33, ns). (**c**) Comparable swimming speed in the Morris water maze (t(18)=1.23, ns).

**Supplementary Figure 4.** (**a**) PDE4B^Y358C/Y358C^ (n=4M/4F) mice demonstrated normal contextual fear at 24 hours compared to PDE4B^+/+^ mice (n=6M/7F), but when retested after 7 days they demonstrate decreased contextual fear (Time F(1,19)=10.77, p<0.01; Genotype F(1,19)=1.17, ns; Genotype*Time F(1,19)=5.16, p<0.05). (**b**) PDE4B^Y358C/Y358C^ mice displayed normal nociception (Cox regression B=-0.53, SE=0.68, Wald=0.62, ns). PDE4B^+/+^ (n=2M/2F) and PDE4B^Y358C/Y358C^ (n=4M/3F) mice were placed in the fear conditioning chambers and footshock current was manually controlled. Beginning at 0.10 mA, footshocks were administered for 10 seconds. If the animal did not vocalize or perform a vertical jump, the animal rested for 30 seconds prior to receiving a shock of greater intensity (0.10 mA increases). (**c**) PDE4B^Y358C/Y358C^ mice do not demonstrate sensorimotor changes in acoustic startle response (PDE4B^Y358C/Y358C^ n=6M/5F vs PDE4B^+/+^ n=9M/9F; t(27)=1.04, ns). (**d**) PDE4B^Y358C/Y358C^ mice do not demonstrate impaired prepulse inhibition. PDE4B^Y358C/Y358C^ mice (n=6M/5F) and PDE4B^+/+^ mice (n=9M/9F) had comparable prepulse inhibition for all prepulse intensities (Genotype: F(1, 54)=0.83, ns; Prepulse F(2, 54)=4.53, p<.05; Genotype*Prepulse (F2, 54)=0.79, ns).

**Supplementary Figure 5.** PDE4B-Y358C mutation does not affect basal synaptic transmission. Input-output function representing presynaptic volley size and corresponding fEPSP slope.
