# Supplementary figures and images for: Specific Inhibition of Phosphodiesterase-4B Results in Anxiolysis and Facilitates Memory Acquisition
Source: Neuropsychopharmacology. 2015 Sep 2;41(4):1080–92. doi: 10.1038/npp.2015.240 (PMC4748432; doi:10.1038/npp.2015.240)

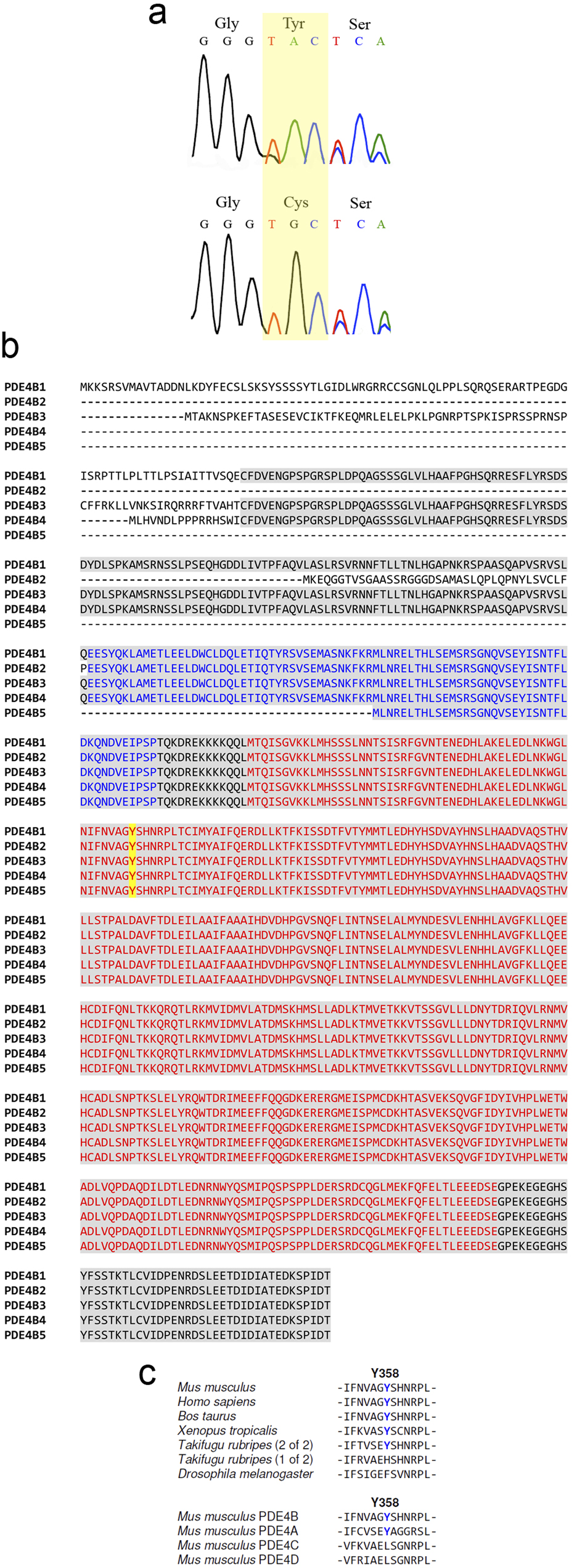

Supplement: Supplementary Figure 1 [file npp2015240x2.tif]

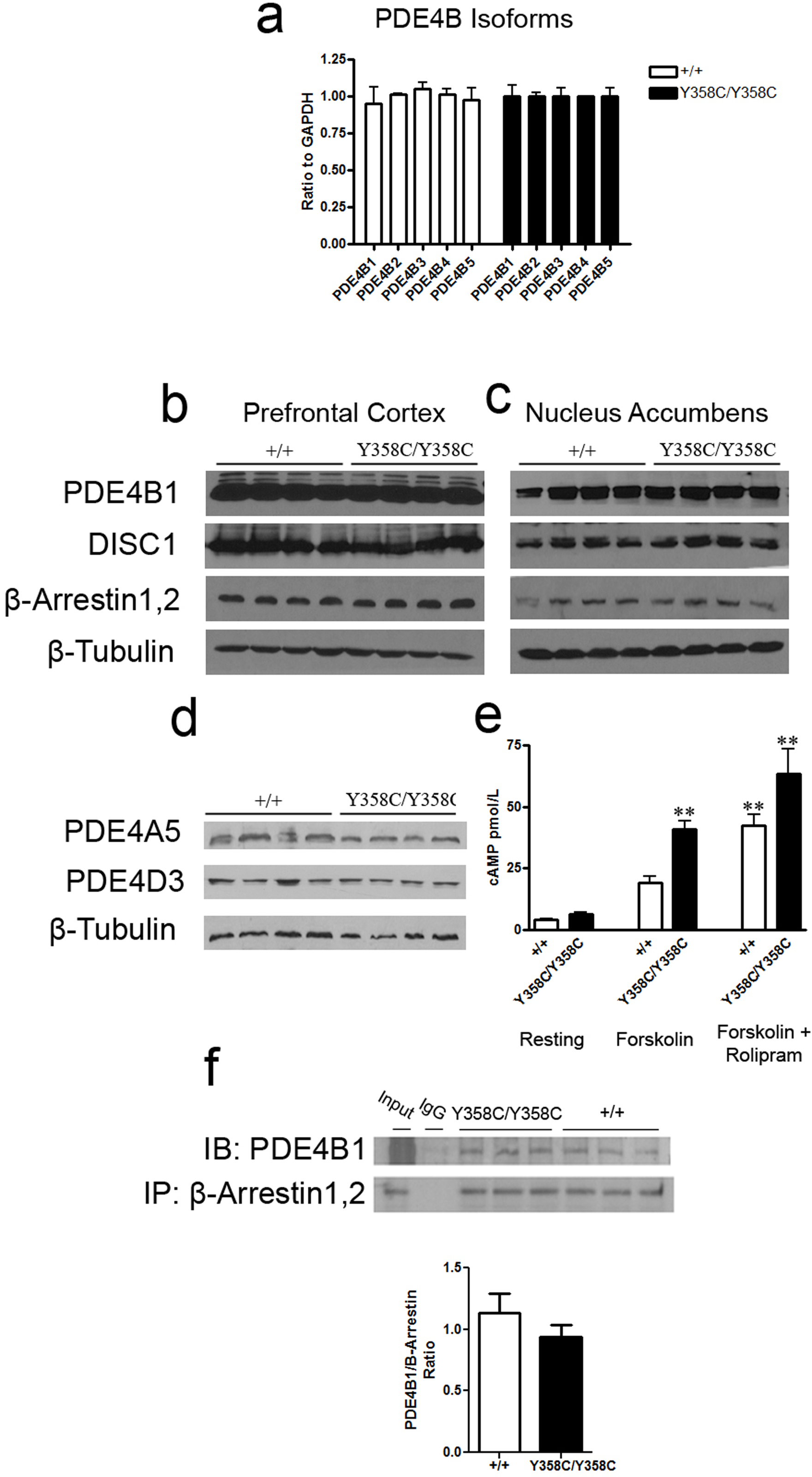

Supplement: Supplementary Figure 2 [file npp2015240x3.tif]

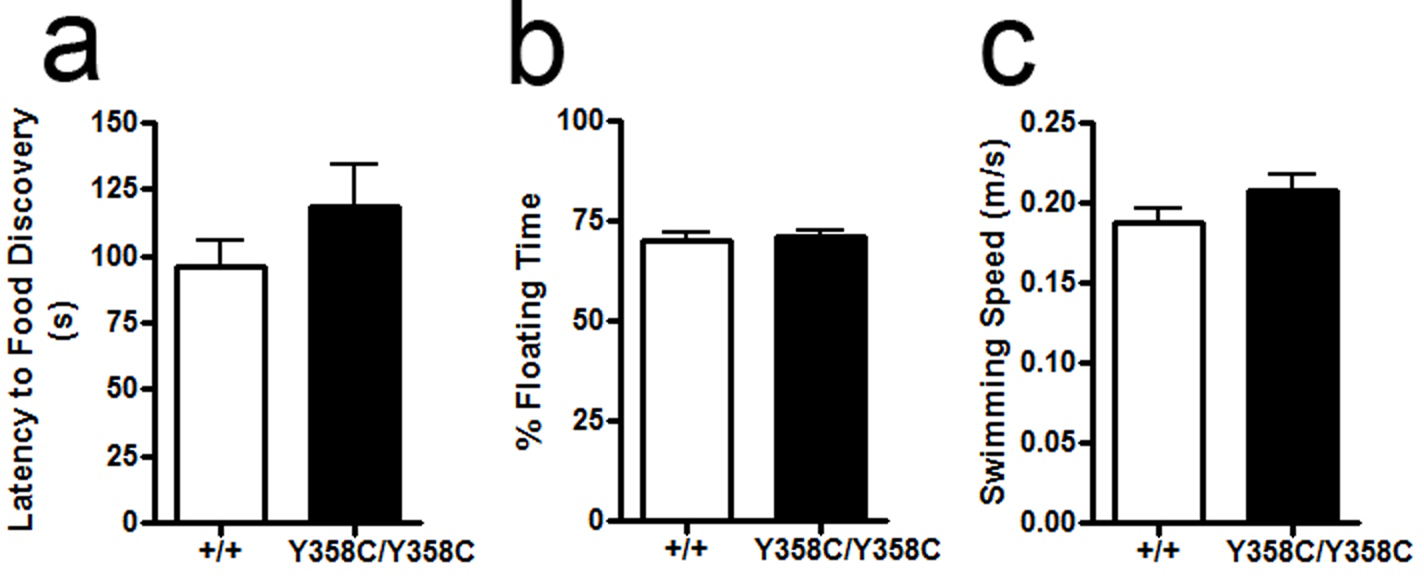

Supplement: Supplementary Figure 3 [file npp2015240x4.tif]

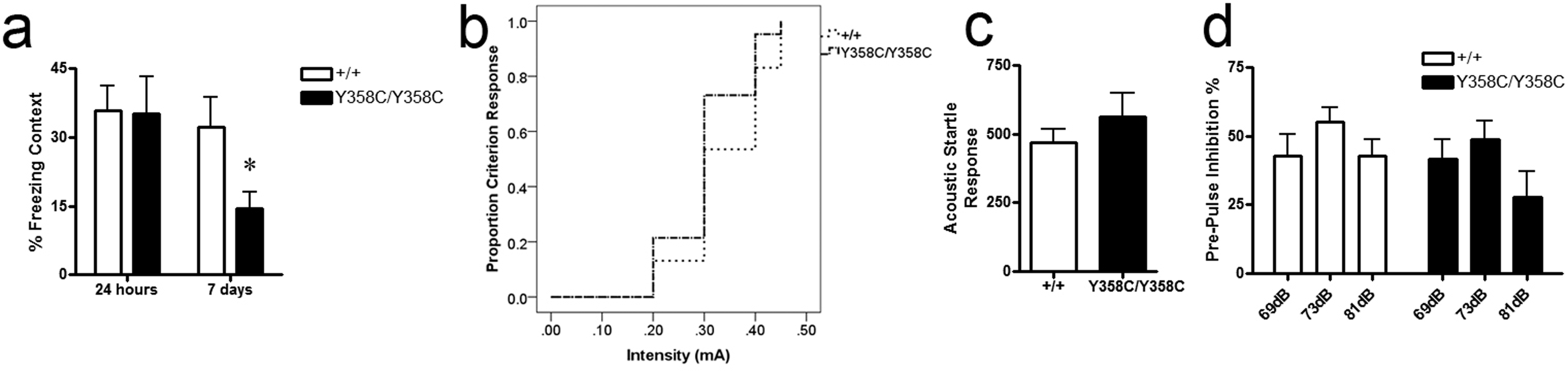

Supplement: Supplementary Figure 4 [file npp2015240x5.tif]

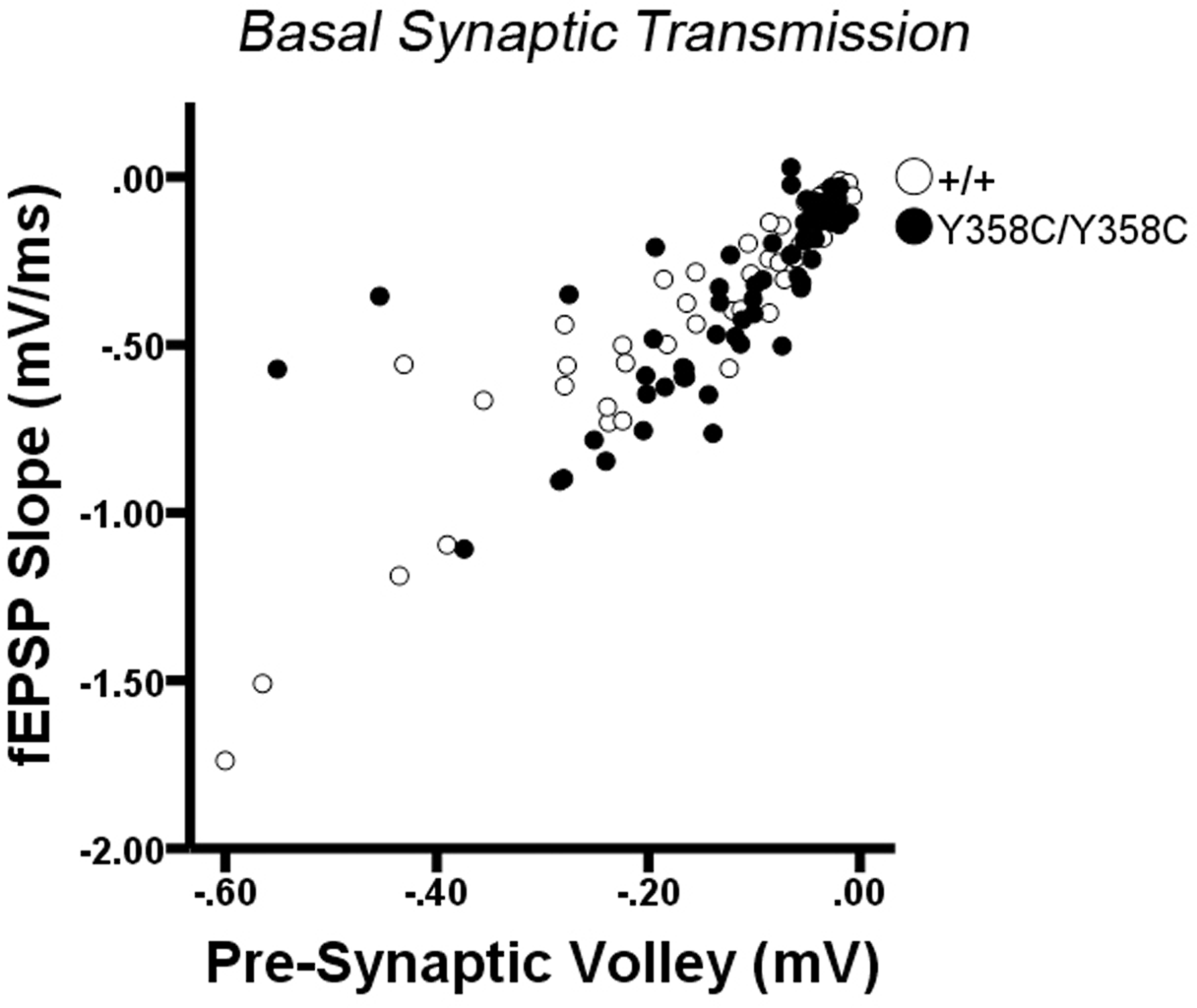

Supplement: Supplementary Figure 5 [file npp2015240x6.tif]
