# Supplementary material for: Specific Inhibition of Phosphodiesterase-4B Results in Anxiolysis and Facilitates Memory Acquisition
Source: Neuropsychopharmacology. 2015 Sep 2;41(4):1080–92. doi: 10.1038/npp.2015.240 (PMC4748432; doi:10.1038/npp.2015.240)
Supplement: Supplementary Methods [file npp2015240x7.doc]

**SUPPLEMENTARY METHODS**

**Mutation Screen**

Heteroduplex detection by high-resolution melting analysis in a LightScanner (Idaho Technology) was used to screen 7776 male F_1_ progeny of ENU-mutagenized BALB/cAnN males and untreated C3H/HeH females for mutations in exon 10 of *Pde4b*, which was amplified using PCR primers F, 5´-TGC ATG AAA GAG TTG GTA GCT AA-3´ and R, 5´-TCT GTT CCA AGA TAA TCG GTG TTT-3́. The A1073G mutation in *Pde4b* was confirmed by DNA sequencing using a BigDye Terminator v3.1 Cycle Sequencing Kit (Applied Biosystems).

**Subjects**

Heterozygous N_2_ backcross progeny of the founder PDE4B^Y358C/+^ (C3H/HeH x BALB/cAnN) F_1_ male (EMRCB/60.3d) and wild-type C57BL/6NTac females (Taconic) were backcrossed through the male and female lines to C57BL/6J (The Jackson Laboratory) for 10 generations before heterozygotes were intercrossed to generate homozygous mutant PDE4B^Y358C/Y358C^ and wild-type PDE4B^+/+^ littermates for phenotypic characterization. The PDE4B^358C^ line was genotyped for the Y358C mutation by the presence of a *Bsi*HKAI (R0570, New England BioLabs) restriction site in a 443-bp fragment amplified using PCR primers F, 5´-ACC TGC CTT TGA AAG TAG CAT-3´ and R, 5´-AGC TCT GTT CCA AGA TAA TCG-3´.

Mice were housed at the Toronto Centre for Phenogenomics (TCP) and studied in accordance with institutional and national guidelines for animal welfare (Province of Ontario Animals for Research Act, 1971 and the Canadian Council on Animal Care). At 3 weeks of age, pups of mixed genotype were weaned and housed by sex in groups of three to five under a 12 hour light/dark cycle (lights on at 07.00). Mice were housed in filtered cages containing corn cob bedding (Bed-O’-Cobs, Andersons), nesting material (Nestlets, Ancare) and *ad libitum* sterile food (2018 Teklad Rodent Diet) and water.

Groups of male and female mice (with the exception of the social recognition test) were tested between 8-12 weeks of age. Testing was conducted between 12h00 and 17h30. Experimenters were blind to genotype, which was determined after data collection. Between mice and after all tests, all apparatus were cleaned with Clidox and 70% ethanol to prevent bias due to olfactory cues. Data collection for most behavioral tests (except where noted) was performed by OBSERVER 5.0 software (Noldus). All animals were characterized in only one behavioural test, with the following exceptions: 1) all animals who completed the elevated plus maze test then also did the open-field test followed by the Y-Maze test, and 2) the exploratory cohort in which test-retest fear conditioning is reported (Supplementary Figure 3a) had been tested on the elevated plus maze, open-field, Y-maze, and the forced swim test prior to undergoing fear conditioning training. Minimum two full days passed between tests in these cases.

**Structural Modeling**

Wild-type and Y358C mutant forms of mouse PDE4B1 were modelled by 98% homology (51% sequence coverage) with a range of PDB templates. Using our multi-template homology modeling pipeline, six PDB homologues were identified and used in the assembly of the PDE4B wild type and Y358C models (1TAZ(chain A); 1SO2(chain D); 1ZKL(chain A); 3G4G(chain D); 3ECM(chain A);1Y2K(chain B)). The binding site of cAMP was predicted by superposition of the mouse PDE4B1 models upon chain A of PDB 1TB5, the catalytic domain of human phosphodiesterase-4B in complex with AMP, using the Chimera program, which was also used for the viewing of models and generation of images. The homology modeling pipeline was built with the Biskit structural bioinformatics platform. Our pipeline workflow incorporates the NCBI tools platform, including the BLAST program for similarity searching of sequence databases. T-COFFEE was used for alignment of the test sequence with the template, followed by iterations of the MODELLER-9.11 program (September 2012 release) to final model structure.

**Mammalian Expression Constructs**

A VSV-epitope-tagged human PDE4B1 cDNA insert was cloned into the *Eco*R1 site of an ampicillin-resistant pEE7 mammalian expression vector (Celltech) as previously described, and was a kind gift from Kirsty Millar (University of Edinburgh). The Y358C mutation was introduced using a QuikChange Site-Directed Mutagenesis Kit (Stratagene) with primers F, 5´-CAT CTT TAA TGT GGC TGG ATC TTC TCA CAA TAG ACC CCT A-3´ and R, 5´-TAG GGG TCT ATT GTG AGA AGA TCC AGC CAC ATT AAA GAT G-3´. The Y358C mutation was confirmed by DNA sequencing using a BigDye Terminator v3.1 Cycle Sequencing Kit (Applied Biosystems).

**Western Blotting**

Brain regions dissected from behaviorally and pharmacologically naïve 8-week-old mice (PDE4B^Y358/Y358C^ n=4, PDE4B^+/+^ n=4; PDE4B^Y358/Y358C^ n=8, PDE4B^+/+^ n=8 for PDE4B1 quantification) were homogenized in RIPA buffer (1 M Tris-HCl, pH 7.5; 3 M NaCl) containing protease inhibitors (Complete Mini, Roche), and then centrifuged at 6,000 rpm for 10 min at 4ºC. Protein was quantified by Bradford assay (Protein Assay, BioRad). Samples were incubated at 95°C for 5 min, and triplicate wells subjected to gradient SDS-PAGE (100 V, 1.5 hr) in 10% Tris-HCl polyacrylamide gels in parallel with 5 µl MagicMark XP Western Protein Standard (Invitrogen), and then blotted (80 V, 1.5 hr) onto nitrocellulose membrane (GE Healthcare). After blocking with 5% milk in PBS-T (PBS, 0.1% Tween-20) for 16 hr, membranes were incubated with primary antibodies – anti-PDE4B1, anti-DISC1 (Santa Cruz), anti-B-Arrestin1,2 (Cell Signaling), anti-PDE4A5 (FabGennix), anti-PDE4D3 (FabGennix) anti-β-Tubulin III (Sigma) – in 1% milk for overnight. Immune complexes were detected by chemiluminescence (ECL-Plus, GE Healthcare) with horseradish peroxidase-conjugated anti-species IgG (GE Healthcare). Membranes were immersed in Restore Western Blot Stripping Buffer (Pierce) before being incubated with the subsequent primary antibody. Densitometry analysis of scanned film, using ImageJ 1.36 software (http://rsb.info.nih.gov/ij), was undertaken to quantify the visualized bands. PDE4B^Y358C/Y358C^ and PDE4B^+/+^immunoreactivity levels are expressed as [mean triplicate signal / mean β-tubulin III signal].

Cell lysate western blots were carried out using the NuPage Novex system (Life Technologies). Primary antibodies were anti-VSV (Sigma), anti-DISC1 (courtesy of Prof Takayama, University of Tokyo) and anti-β-Arrestin1,2 (Santa Cruz Biotechnology). Secondary antibodies were IRDye 800CW and IRDye 680RD antibodies (LICOR). Densitometry analysis was performed using LICOR Odyssey system and quantified using LICOR image studio.

**Immunoprecipitation**

HEK-293 cells transfected with VSV-PDE4B1-WT and VSV-PDE4B1-WT constructs were lysed on ice. Lysates were normalized to 1mg/ml by Bradford assay. Using 400μg of lysate, tagged proteins were pulled down using monoclonal anti-VSV-glycoprotein-agarose (Sigma), and negative controls pulled down using anti-FLAG M2 affinity gel (Sigma). Using anti-DISC1 antibody (courtesy of Prof. Takayama, University of Tokyo), we then probed the antigen.

**Co-Immunoprecipitation**

Whole mouse brains of behaviorally and pharmacologically naïve mice were homogenized in RIPA buffer containing 50 Mm Tris-Cl, pH 7.6, 150 mM NaCl, 2 mM EDTA, 1 mM PMSF plus 1% Igepal CA-630, 0.5-1% sodium deoxycholate, 1% Triton X-100, and protease inhibitor mixture (5 μl/100 mg of tissue; Sigma). After centrifugation at 10,000 × *g* at 4°C for 20 min, the supernatant was extracted and protein concentrations were measured. Twenty μl of protein A/G agarose (Santa Cruz Biotechnology) were washed once with RIPA buffer. Solubilized mouse brain tissues (500 μg DISC1, 2 mg β-Arrestin) were pre-incubated with 20 μl of protein A/G agarose for 1 h at 4°C to reduce non-specific binding. The beads were incubated in the presence of primary antibodies, anti-DISC1 (Santa Cruz Biotechnology), anti-β-Arrestin (Santa Cruz Biotechnology) or rabbit IgG (A0545, Sigma, 1-2 μg), in RIPA buffer for 4 h at 4°C followed by the addition of solubilized whole brain extracts. The mixture was then incubated for 12 h at 4°C with gentle rotation. Pellets were washed four times in the RIPA buffer each time for 5 min. Samples were boiled for 5 min at 100°C in 20 μl 2 x SDS sample buffer or incubated at 37°C for 45 min to move the IgG band up to 100 kDa, and then subjected to SDS-PAGE. Fifty μg of tissue-extracted protein was used as a positive control. Proteins were then transferred electrophoretically to a 0.2 μm nitrocellulose membrane. Nonspecific binding sites were blocked by incubating the blots in 5% non-fat powdered milk for 1 h at room temperature and incubated with an appropriate primary antibody, anti-PDE4B1), at 4°C overnight with a gentle rotation. Total PDE4B1 bound to DISC1 as well as direct DISC1 immunoprecipitation levels were determined using horseradish peroxidase-linked secondary antibodies and enhanced chemiluminescence detection. The intensity of each protein band was quantified by densitometry using ImageJ software.

**PDE4B Isoform Quantification:RNA Isolation and Real-Time PCR Analysis.**

Mice were euthanized by cervical dislocation and hippocampus was dissected in ice-cold PBS for RNA extraction. RNA was isolated using Trizol (Invitrogen^TM^ Life Technologies, Carlsbad, CA, USA) according to the manufacturer’s specifications. cDNA was generated using Reverse transcriptase III (Invitrogen^TM^ Life Technologies). Real-time polymerase chain reaction (PCR) performed using SYBR Green (Qiagen) and ABI prism and SDS 2.1 software (AppliedBiosystems Inc., Foster City, CA, USA). PDE4B splice variant 1-5 and Gapdh mRNA expression level was measured. Quantitative PCR were run in triplicate and threshold cycle (Ct) values averaged. Data were then normalized to *Gapdh*. A region of the PDE4B splice variant 1-5 and*Gapdh*mRNA was amplified using primers as previously described .

**PDE4B Function**

PDE activity was determined using a two-step radioassay procedure as described previously with lysates from VSV-PDE4B1-WT and VSV-PDE4B1-Y358C transfected HEK-293 cells.

**Drugs**

Forskolin and rolipram (Enzo Life Sciences, Farmingdale, NY) were kept in frozen (-30C) stock solutions in DMSO. Drugs were administered at a final volume of 10ml/kg. Final DMSO concentration was for in vivo experiments was Rolipram 1.25%, BrdU 5%, and 1% for in vitro experiments.

**Transfection**

HEK293 cells were maintained in DMEM with 10% FBS at 37◦C and 5% CO2. Cells were seeded into 6 well plates and once 80% confluent, transfected with 14μL lipofectamine (Invitrogen) and 14μg DNA for 24 hours.

**cAMP Quantification**

Hippocampal transverse slices (400μm) assayed for cAMP accumulation according to three conditions: 1) resting slices, 2) slices incubated in 10μM forskolin for 15 min, 3) slices incubated in 70 μM rolipram for 15 min, and 4) slices incubated in 10μM forskolin and 70μM rolipram for 15min. Slices were transferred into eppendorf tubes and frozen in liquid nitrogen. After adding100 µl of 5% TSA, the tissue was homogenized and cAMP quantified according to the manufacturer’s instructions (Cyclic AMP EIA Kit, Cayman).

**Open Field Test**

The activity arena (42cm x 42cm x 30cm) was equipped with horizontal and vertical infrared sensors (Accuscan Instruments Inc, Ohio, USA). The chamber of the test arena was illuminated at 500 lux. Each mouse was placed individually into the center of the activity cage and motor activity measured during the 30 minute test period. We report the total distance traveled, time in seconds spent in the centre of the arena and periphery of the arena and the number of rearing movements.

**Elevated Plus Maze (EPM)**

Animals were tested in a dimly lit room, with a bright light (210 lux) focused on the central platform. The elevated plus maze consisted of two closed arms (25x5x30cm) and two open arms (25x5cm) with an open centre platform (5x5cm). Animals were placed in the central platform, after which they were observed for 5 minutes. Entries were defined as four paws entering an arm, and time spent in the open arms, closed arms, and centre were coded separately. Risk assessment was defined as exploration with the head at the level of the tragus exiting the dark arm. Passages are defined as crossing the central platform without turning. Head dips were defined as the tragus being level with the platform. The data is presented as counts (passages & head dips) and percentage of the testing period (light arm & dark arm).

**Light/dark Transition Test**

The apparatus consisted of a cage (42×42×30cm) divided into two chambers of equal size, one bright and one dark. The partition separating these sections contained a door allowing free movement between the sections. Mice were placed in the dark chamber and the time spent in the light and dark chambers was recorded over a 5 minute observation period. Entry into the light chamber was defined as four paws crossing the door. The data is presented as percentage of time spent in the light chamber.

**Cat Urine Fear Assessment**

In a T-shaped maze (65 cm x 14 cm long arm, 30 cm x 14 cm short arms), mouse chow was placed at the far end of a short arm and a piece of cloth with three drops of bobcat urine (Maine Outdoor Solutions; [www.predatorpee.com](http://www.predatorpee.com/)) was placed at the far end of the opposite arm. The maze was placed within a fume hood. Mice were allowed 5 minutes to explore the maze, and the number of entries into each arm was recorded and presented as % of the total number of entries.

**Forced Swim Test**

Individual mice were placed into a transparent glass beaker (25 cm height, 18 cm diameter), containing water at 25°C. For 6 minutes, the mice remained in the water while an observer coded their active swimming and floating (minimal efforts to keep head above water). The water was changed between subjects. Only the last 4 minutes of the experiment are reported.

**Hole Board Test**

Testing took place in a Plexiglas arena (40cm x 40 cm, 30 cm in height) in which 9 circular holes (radius 2cm) are organized in octagonal shape around a central hole. The arena was raised 8cm above a clean surface and dimly lit (210 lux). Mice were observed in the arena for 5 minutes. Hole pokes were defined as an investigation of the hole with the nose traversing the thickness of the Plexiglas.

**Y-Maze**

Testing took place in a three pronged maze with arms (40cm x 8cm x 15cm) at 120 degrees from one another. The walls of each arm are adorned with distinctive geometric shapes. Mice begin the testin one arm and each successive mouse is placed in a different arm to avoid arm preference. Leaving an arm was defined as all four paws entering the hexagonal centre. Arm entries were defined as all four paws entering the arm. Number of entries is recorded. The first arm is not scored. Spontaneous alternation refers to visiting all three arms in sequence (spontaneous alternating sequences/(total arms -2) x 100), alternative arm alternation refers to re-entering an arm after having visited another (alternating arm sequences/(total arms -2) x 100), while same arm re-entry is leaving an arm only to return immediately (re-entries/(total arms-2) x100).

**Morris Water Maze**

Mice were tested in a cylindrical tank of 185 cm diameter with water (26±1°C) made opaque with white non-toxic paint. Visual cues adorned the walls of the test room. A camera above the pool center recorded mice in the water and the video output was analysed in automated fashion (HVS Water 2020; HVS Image). Each experimental protocol began with three training trials on a single day with a stationary 10-cm visible platform placed 15cm from the tank edge and 0.5cm above water. For the four subsequent days, the platform was hidden in the same position by submerging it 1 cm below the surface of the opaque water. Each day, mice performed three trials with pseudo-random start points at 1hr inter-trial intervals. Mice that found the platform were left on it for 15s, while mice unsuccessful after 90s were placed on the platform for 15s. Reversal learning took place with a displaced platform for three training days. Probe trials involved removing the platform from the tank and quantifying the amount of time spent in each of the quadrants during a the first 30s of the testing period. Probe trials took place 24 hours after the conclusion of training. Data are presented as latency to reach the platform and were analysed by one-way ANOVA (genotype as a main factor) with repeated measures and time in quadrant with one-way ANOVA. Post-hoc comparisons were followed by Fisher’s least significant difference (LSD) test.

**Object Location Recognition**

Experiments were conducted in a square (40cm x 40 cm, 30 cm in height) clear Plexiglas arena that was brightly illuminated (500 lux). Mice were initially habituated to the environment, in which objects had been placed in the four corners of the arena, and then returned to the home cage for 3 minutes. To test spatial memory, mice were reintroduced and left for 5 minutes in the arena, in which two of the four objects had now been displaced to the center. To test the emotional modulation of spatial memory acquisition, we created a threatening environment by suspending the clear Plexiglas arena 1 m above the room floor and placing a black and white check pattern (16 cm^2^ checkers) on the floor to accentuate the height. Acquisition occurred for 5 or 10 minutes with either a ‘safe’ or ‘threatening’ environment.

**Social Recognition**

Adult male mice were introduced to a plexiglass cage without bedding (30×17×12cm) and allowed to explore for 5 minutes, after which a male juvenile was introduced for a 2 minute interaction trial. The cage was cleaned with 70% ethanol between subjects. 24hrs later, the adult was habituated to the testing cage prior to the reintroduction of the familiar juvenile for 2 minutes. Two hours later, a novel juvenile male was introduced for 2 minutes. Anogenital investigation was recorded.

**Contextual and Cued Fear Conditioning**

Contextual and cued fear conditioning experiments were performed in sound-attenuated chambers (Med Associates) equipped with video cameras placed outside the chamber, and a computer-controlled fear conditioning system (Actimetrics). Mice were placed in the chambers and allowed to explore for 2 minutes prior to beginning the training session. The training session consisted of two additional minutes of recorded exploration, followed by an auditory cue (3600Hz, 80dB) for 30 seconds, which in turn was immediately followed by a foot shock (1 mA) of 2 seconds duration.

After 24 hours (or 7 days), contextual fear memory was assessed by returning the mice to the training chamber and evaluating exploration for 5 minutes. Two hours later, cued fear memory was evaluated by returning the mice to the training chamber that had been modified (flooring with solid opaque plastic, chamber configuration by including a prism tent, visual cues with vertical stripes, and scent with 3% acetic acid) for 3 minutes of exploration prior to presenting the cue for an additional 3 minutes. Results are presented as the percentage of time the animal engaged in freezing behavior, defined as the complete absence of any movement except for respiration and heartbeat. This was measured during the context and cued conditioning tests at 0.25 second intervals by using FreezeFrame automated fear conditioning software (Actimetrics).

A separate cohort of PDE4B^+/+^ mice underwent fear conditioning and were then given twice daily intraperitoneal injections of rolipram 1mg/kg from 24 hours to 6 days following conditioning. On day 7, at least 15 hours after the final rolipram injection, mice were tested for contextual and cued fear memory as described above. Mice tested for fear conditioning were excluded from subsequent behavioral, biochemical or electrophysiological testing.

**Subchronic Rolipram Following Pavlovian Fear Conditioning**

PDE4B+/+ mice underwent fear conditioning as described below. Following a 24hr delay to allow encoding of long-term memory, twice daily intraperitoneal injections of rolipram 1mg/kg were administered from 24 hrs-6 days following conditioning. On day 7, a minimum of 15 hrs following the final rolipram injection (5 t_1/2_), mice were tested for contextual and cued fear memory as described above.

**Footshock Pain Threshold**

Naïve mice were placed in the fear conditioning chambers and left to habituate to the chamber for 5 minutes. Then, beginning at 0.10 mA, footshocks were administered for 10 seconds. If the animal did not vocalize or perform a vertical jump exceeding resting height, the animal rested for 30 seconds prior to receiving a shock of greater intensity (0.10 mA intervals). The intensity at which mice vocalized or performed a vertical jump exceeding the animal’s height was recorded. Data were analyzed using Cox regression and the survival function is presented.

**Olfactory Testing**

Mice were first habituated to food (Bud's Best Cookies, Hoover, AL, USA) using food pellets (1×1×0.5cm) left overnight in the home cage. Mice were subsequently food deprived for 24 hours. The test was conducted in a cage similar to the home cage (30×17×12cm), in which a pellet was placed in a randomly chosen area and then the entire cage floor (including pellet) was covered with corncob bedding to a depth of 2.5cm. The mouse was then placed into the cage and latency to find the food was recorded up to a maximum time limit of 15 min. Latency to food discovery and total time burrowing were recorded.

**Prepulse Inhibition (PPI) of Acoustic Startle Response**

PPI testing was conducted in sound damped isolation chambers in which animals are confined to a holder (Med. Associates Inc., Startle Reflex System, St Albans, VT). All events were recorded and controlled by Med Associates software (Startle Reflex package). Background noise was set at 65dB. Testing involves: 1) startling pulse trials consisting of a white noise burst (120dB, 40ms); 2) prepulse + pulse trials consisting of a prepulse of noise (20ms at 69, 73, or 81dB, respectively) with a startling pulse (120dB, 40 ms) 100 ms later; 3) no-stimulus trials consisting of background noise only. Mice are initially acclimatized to the background noise for 15 minutes, presented with 5 startling pulse trials, then 10 blocks each containing 5 pseudorandomly ordered prepulse intensities, and an additional 5 startle pulses at the conclusion. The force intensity for each trial was recorded as the startle level. The percentage PPI induced by each prepulse intensity was calculated as [1-(startle amplitude on prepulse trial)/(startle amplitude on pulse alone)] × 100%. Startle amplitude in this formula was calculated as the average response to all of the pulse alone trials, excluding the first and last of five pulse alone trials.

**Electrophysiology in CA1 of Hippocampal Slices**

Adult mice (8-12 weeks; high-frequency stimulation experiments) or post-natal day 16-17 mice (low-frequency stimulation experiments) were anesthetized, decapitated and their brains quickly removed into ice-cold artificial cerebral spinal fluid (ACSF) consisting of: 124 mM NaCl, 26 mM NaHCO_3_, 10 mM d-glucose, 3 mM KCl, 2 mM CaCl_2_, 2 mM MgSO_4_, and 1.3 mM NaH_2_PO_4_ saturated with 95% O_2_–5% CO_2_. Both hippocampi were removed and, using a tissue chopper, 400 μm hippocampal slices were prepared and placed in a holding chamber at room temperature for at least 2 h before use.

Electrophysiological recordings were performed in a perfusion-style chamber with ACSF maintained at 30°C with a heat controller and fresh ACSF provided with a mini-pump. For all experiments, with the exception of forskolin experiments, the above described ACSF was perfused. For experiments using forskolin, an ACSF solution with reduced MgSO_4_ concentration (0.75 mM) was used. Glass microelectrodes containing ASCF without Ca^2+^ (3 MΩ resistance) were used to record extracellular field potentials.

Responses were collected every 10s, amplified and filtered at 2-kHz using an Axopatch 1D, and then digitized at 10-kHz using pCLAMP9 software (Axon Instruments). For all experiments, glass recording micropipettes were positioned in the hippocampal CA1 stratum radiatum region to record activity evoked by stimulation (tungsten electrodes) of Schaffer collateral axons. Stimulus intensity was adjusted to yield population excitatory post-synaptic potentials (fEPSPs) that were 40% of the maximal spike-free size. The slope of each fEPSP was calculated between 10-50% of the peak. When plotting results, the slope of fEPSPs was normalized to the mean slope obtained during the 15min baseline prior to tetanus. Each data point reflects an average of six responses (60 s); only one data point every minute is shown.

For forskolin experiments, after reaching a stable baseline, reduced magnesium-ACSF with 10 μM of forskolin (from 10 mM stock in DMSO; final DMSO concentration 0.1%) was perfused for 10 minutes. With respect to LTP experiments, tetanic stimulation was delivered in the form of four trains (500 ms duration, 10 s apart) at 100-Hz. For brief tetanic stimulation, stimulation was delivered in the form of a single train (500 ms duration) at 100-Hz. Low frequency stimulation experiments involved 1-Hz for 15 minutes (900 pulses). For depotentiation experiments, tetanic stimulation (4 x 100-Hz, 500 ms, 10 s apart) was delivered and the fEPSPs allowed to stabilize for 10 minutes prior to receiving low frequency stimulation (1-Hz) for 15 minutes (900 pulses). Paired-pulse facilitation (PPF) was tested by delivering stimuli in succession at less than 1 s intervals. The inter-trial interval was 10 s, with progressively smaller pair-pulse interval. Facilitation is calculated as the ratio of the fEPSP slope of the second paired pulse over the slope of the first paired pulse.

**Dendritic Spine Density**

*Thy1*-GFP mice were acquired from The Jackson Laboratory. PDE4B^Y358/Y358C^ mice were crossed with *Thy1*-GFP mice to produce PDE4B^Y358C/+^;*Thy1*-GFP mice, which were intercrossed to generate PDE4B^Y358/Y358C^;*Thy1*-GFP and PDE4B^+/+^;*Thy1*-GFP mice for analysis. At 8 weeks, these mice were anesthetised and transcardially perfused with heparinised saline followed by 4% paraformaldehyde (PFA). The brains were removed and placed overnight in 4% PFA. They were transferred to 40% sucrose solution for 3 days prior to being frozen and, using a cryostat, coronally sliced at 70μm thickness. Hippocampal CA1 and lateral amygdala neurons were imaged by confocal microscopy (Nikon Eclipse C1si). Dry and oil immersion lenses were used to identify and acquire images using 1 μm z-stacks. Images were analysed using Nikon Elements software. Second order branches were identified and spines counted over 10-15 μm segments from independent neurons. Results are presented as mean ± S.E.M. densities.

**Neurogenesis**

Twenty four hours after fear conditioning, mice received daily injections of 50 mg/kg of BrdU (Sigma) for four days. Mice were then retested for contextual and fear conditioning at day 7, after which they were anesthetised and transcardially perfused with heparinised saline followed by 4% paraformaldehyde. Brains kept overnight in 4% paraformaldehyde before being placed in 40% sucrose for 3 days. They were then frozen and coronal slices 50μm thick cut using a cryostat. Once hippocampal tissue was identified, every 8^th^ slice was retained for staining and analysis. DNA was denatured with 2M hydrochloric acid for 25 minutes prior to washing with phosphate buffered saline. Slices were then incubated with anti-doublecortin and anti-BrdU (Santa Cruz Biotechnology). Slices were imaged by confocal microscopy (Nikon Eclipse C1si). Images were analysed using Nikon Elements, the length of subgranular zone characterized wastraced and measured, and BrdU positive and doublecortin positive cells counted.

**References**

Crawley J, Goodwin FK (1980). Preliminary report of a simple animal behavior model for the anxiolytic effects of benzodiazepines. *Pharmacol Biochem Behav* **13**(2): 167-170.

Engelmann M, Hadicke J, Noack J (2011). Testing declarative memory in laboratory rats and mice using the nonconditioned social discrimination procedure. *Nat Protoc* **6**(8): 1152-1162.

Eswar N, John B, Mirkovic N, Fiser A, Ilyin VA, Pieper U*, et al* (2003). Tools for comparative protein structure modeling and analysis. *Nucleic Acids Res* **31**(13): 3375-3380.

Feng G, Mellor RH, Bernstein M, Keller-Peck C, Nguyen QT, Wallace M*, et al* (2000). Imaging neuronal subsets in transgenic mice expressing multiple spectral variants of GFP. *Neuron* **28**(1): 41-51.

File SE, Wardill AG (1975). The reliability of the hole-board apparatus. *Psychopharmacologia* **44**(1): 47-51.

Geyer MA, Dulawa SC (2003). Assessment of murine startle reactivity, prepulse inhibition, and habituation. *Curr Protoc Neurosci* **Chapter 8**: Unit 8 17.

Gobejishvili L, Avila DV, Barker DF, Ghare S, Henderson D, Brock GN*, et al* (2011). S-adenosylmethionine decreases lipopolysaccharide-induced phosphodiesterase 4B2 and attenuates tumor necrosis factor expression via cAMP/protein kinase A pathway. *J Pharmacol Exp Ther* **337**(2): 433-443.

Grunberg R, Nilges M, Leckner J (2007). Biskit--a software platform for structural bioinformatics. *Bioinformatics* **23**(6): 769-770.

Hall C, Ballachey EL (1932). A study of the rat's behavior in a field: a contribution to method in comparative psychology. *Unjiversity of California Publications in Psychology* **6**: 1-12.

Hughes RN (2004). The value of spontaneous alternation behavior (SAB) as a test of retention in pharmacological investigations of memory. *Neurosci Biobehav Rev* **28**(5): 497-505.

Huston E, Lumb S, Russell A, Catterall C, Ross AH, Steele MR*, et al* (1997). Molecular cloning and transient expression in COS7 cells of a novel human PDE4B cAMP-specific phosphodiesterase, HSPDE4B3. *Biochem J* **328 ( Pt 2)**: 549-558.

Johansson EM, Sanabra C, Cortes R, Vilaro MT, Mengod G (2011). Lipopolysaccharide administration in vivo induces differential expression of cAMP-specific phosphodiesterase 4B mRNA splice variants in the mouse brain. *J Neurosci Res* **89**(11): 1761-1772.

Leger M, Quiedeville A, Bouet V, Haelewyn B, Boulouard M, Schumann-Bard P*, et al* (2013). Object recognition test in mice. *Nat Protoc* **8**(12): 2531-2537.

Marchmont RJ, Houslay MD (1980). A peripheral and an intrinsic enzyme constitute the cyclic AMP phosphodiesterase activity of rat liver plasma membranes. *Biochem J* **187**(2): 381-392.

Notredame C, Higgins DG, Heringa J (2000). T-Coffee: A novel method for fast and accurate multiple sequence alignment. *J Mol Biol* **302**(1): 205-217.

Pettersen EF, Goddard TD, Huang CC, Couch GS, Greenblatt DM, Meng EC*, et al* (2004). UCSF Chimera--a visualization system for exploratory research and analysis. *J Comput Chem* **25**(13): 1605-1612.

Porsolt RD, Bertin A, Jalfre M (1977). Behavioral despair in mice: a primary screening test for antidepressants. *Arch Int Pharmacodyn Ther* **229**(2): 327-336.

Stephens PE, Cockett MI (1989). The construction of a highly efficient and versatile set of mammalian expression vectors. *Nucleic Acids Res* **17**(17): 7110.

Vorhees CV, Williams MT (2006). Morris water maze: procedures for assessing spatial and related forms of learning and memory. *Nat Protoc* **1**(2): 848-858.

Walf AA, Frye CA (2007). The use of the elevated plus maze as an assay of anxiety-related behavior in rodents. *Nat Protoc* **2**(2): 322-328.

Wehner JM, Radcliffe RA (2004). Cued and contextual fear conditioning in mice. *Curr Protoc Neurosci* **Chapter 8**: Unit 8 5C.

Wheeler DL, Barrett T, Benson DA, Bryant SH, Canese K, Chetvernin V*, et al* (2008). Database resources of the National Center for Biotechnology Information. *Nucleic Acids Res* **36**(Database issue): D13-21.

Yang M, Crawley JN (2009). Simple behavioral assessment of mouse olfaction. *Curr Protoc Neurosci* **Chapter 8**: Unit 8 24.

Zhang KY, Card GL, Suzuki Y, Artis DR, Fong D, Gillette S*, et al* (2004). A glutamine switch mechanism for nucleotide selectivity by phosphodiesterases. *Mol Cell* **15**(2): 279-286.
